# Cortical processing during robot and functional electrical stimulation

**DOI:** 10.3389/fnsys.2023.1045396

**Published:** 2023-03-21

**Authors:** Woosang Cho, Carmen Vidaurre, Jinung An, Niels Birbaumer, Ander Ramos-Murguialday

**Affiliations:** ^1^Institute of Medical Psychology and Behavioral Neurobiology, University of Tübingen, Tübingen, Germany; ^2^g.tec Medical Engineering GmbH, Schiedlberg, Austria; ^3^TECNALIA, Basque Research and Technology Alliance, Neurotechnology Laboratory, San Sebastián, Spain; ^4^Ikerbasque-Basque Foundation for Science, Bilbao, Spain; ^5^Interdisciplinary Studies, Graduate School, Daegu Gyeongbuk Institute of Science and Technology, Daegu, Republic of Korea; ^6^San Camillo Hospital, Institute for Hospitalization and Scientific Care, Venice Lido, Italy

**Keywords:** somatosensory mu rhythm, robot, functional electrical stimulation, kinematic, kinetic, neurorehabilitation

## Abstract

**Introduction:**

Like alpha rhythm, the somatosensory mu rhythm is suppressed in the presence of somatosensory inputs by implying cortical excitation. Sensorimotor rhythm (SMR) can be classified into two oscillatory frequency components: mu rhythm (8–13 Hz) and beta rhythm (14–25 Hz). The suppressed/enhanced SMR is a neural correlate of cortical activation related to efferent and afferent movement information. Therefore, it would be necessary to understand cortical information processing in diverse movement situations for clinical applications.

**Methods:**

In this work, the EEG of 10 healthy volunteers was recorded while fingers were moved passively under different kinetic and kinematic conditions for proprioceptive stimulation. For the kinetics aspect, afferent brain activity (no simultaneous volition) was compared under two conditions of finger extension: (1) generated by an orthosis and (2) generated by the orthosis simultaneously combined and assisted with functional electrical stimulation (FES) applied at the forearm muscles related to finger extension. For the kinematic aspect, the finger extension was divided into two phases: (1) dynamic extension and (2) static extension (holding the extended position).

**Results:**

In the kinematic aspect, both mu and beta rhythms were more suppressed during a dynamic than a static condition. However, only the mu rhythm showed a significant difference between kinetic conditions (with and without FES) affected by attention to proprioception after transitioning from dynamic to static state, but the beta rhythm was not.

**Discussion:**

Our results indicate that mu rhythm was influenced considerably by muscle kinetics during finger movement produced by external devices, which has relevant implications for the design of neuromodulation and neurorehabilitation interventions.

## 1. Introduction

Electroencephalography (EEG) and magnetoencephalography (MEG) acquire brain activity’s electrical and magnetic fields, respectively. They can be categorized by activated cortical topography and oscillatory frequency components responding to specific intrinsic and extrinsic causes. The alpha rhythm is one of the most well-known oscillations among them. It is prominently found over the occipital cortex in the absence of visual stimulus in closed eyes, but its amplitude is suppressed while visual input is present. Similar phenomena were observed during the somatosensory stimulation, known as mu rhythm. Both mu and alpha rhythms share common characteristics, such as reflecting the idle state and tuning to be ready for the upcoming input (Kuhlman, 1978). However, they are considered spatially and functionally independent because visual stimuli do not significantly influence the mu rhythm, and somatosensory stimuli do not considerably affect the alpha rhythm either (Kuhlman, 1978). While the mu rhythm is suppressed in response to the sensorimotor tasks, the blood-oxygen-level-dependent (BOLD) signal in functional magnetic resonance imaging (fMRI) increased (Ritter et al., 2009). Also, the gamma rhythm in EEG (Ball et al., 2008; Wagner et al., 2019) is enhanced, and neuron spike rates (Miller et al., 2007; Klimesch, 2011) in invasive recordings increased during the sensorimotor tasks. Its inverse correlation with fMRI, EEG (oscillatory activity in other frequency bands), and neural recordings provided evidence that the suppressed mu rhythm implies cortical excitability, and the enhanced mu rhythm infers cortical inhibition. The enhanced/suppressed features of mu- and beta-rhythm are used for clinical application to detect the motor intention to control the external devices for assistive or motor rehabilitation devices. However, the neural mechanisms and origins of the enhanced/suppressed SMR have not been clearly explained in terms of psychological and physiological conditions. The level of enhancement/suppression varies within and between subjects; even no meaningful changes were detected in some people (Guger et al., 2003). The importance of the sensory role has not been emphasized in SMR research and clinical application. The SMR in response to different proprioceptive stimulation has not been widely studied.

Despite inconsistency in the terms and range of its frequency bands in the literature, sensorimotor rhythm (SMR) can be classified into two oscillatory frequency components: mu band and beta band (Hari and Salmelin, 1997; Jones et al., 2009). The beta band (14–25 Hz with a central frequency of around 20 Hz) had been regarded as nothing more than the harmonic rhythms of the mu band (8–13 Hz with a central frequency of around 10 Hz). Still, more evidence of functional and topographical differences has been uncovered. The lower frequency component is localized in the post-central somatosensory cortex, and the higher frequency component is located in the pre-central motor cortex (Pfurtscheller et al., 1994; Salmelin and Hari, 1994; Salmelin et al., 1995; Hari and Salmelin, 1997). In addition, the correlation with EMG and the time of recovery-to-baseline after events were different from each other (Stancák and Pfurtscheller, 1995). Therefore, they are hereafter referred to as mu and beta, respectively, to emphasize the separate frequency components of the oscillation. Both rhythms have been used to study sensorimotor processing (Birbaumer et al., 1990; Neuper et al., 2006; Buzsáki, 2009; Ramos-Murguialday and Birbaumer, 2015), and their suppression and enhancement in power are commonly known as event-related desynchronization (ERD) and event-related synchronization (ERS) (Pfurtscheller et al., 1997, 1998; Houdayer et al., 2006).

The somatosensory information plays a role beyond just providing sensory input. In a recent ECoG study, the primary somatosensory cortex (S1) was activated before the motor cortex activation in cued finger movements, which signifies that sensory information is involved in movement anticipation (Sun et al., 2015). Recent brain-machine interface (BMI) studies successfully decoded upper limb movements from S1 in paralyzed and amputated patients (Wang et al., 2013; Kikkert et al., 2016; Degenhart et al., 2018; Ramsey, 2019; Vidaurre et al., 2019b). The sensory evoked potential (SEP) confirms the arrival to the cortex, proving the intact sensory pathway, as an evoked response in short-lasting stimulation (e.g., tactile stimuli). However, the realistic somatosensory stimuli last a few seconds when related to multi-joint movement as a non-phase-locked response. Therefore, the somatosensory mu and beta ERD/ERS may be more appropriate than SEP in assessing cortical activation in response to movement.

When it comes to brain-body interactions, SMR is correlated with the kinematics (speed, velocity, and acceleration) and kinetics (muscles and force) of limb movement (Yuan et al., 2010; Bourguignon et al., 2019; Branco et al., 2019). In cortico-kinematic coherence (CKC) studies, S1 activity was highly correlated with hand kinematics, reflecting that the cortical processing was driven by movement rhythmicity (Bourguignon et al., 2015). However, one has to be careful regarding the methods used (Antelis et al., 2013). In line with the CKC studies, the mu and beta ERD during dynamic conditions were greater than those during static conditions (Nakayashiki et al., 2014). Different kinetic conditions of muscle and forces also influence the amplitude of the SMR, even at the head movements level (Bibián et al., 2021). Corticomuscular coherence (CMC) studies showed that beta rhythm was correlated with electromyography (EMG) activity, mainly reflecting efferent information (Chakarov et al., 2009; Bourguignon et al., 2019; Vidaurre et al., 2019a; Kenville et al., 2020).

Recently, interventions using multiple body actuating devices, such as the neurofeedback training in control of robotic devices and FES (Grimm and Gharabaghi, 2016; Liu et al., 2017), have attracted interest. Rehabilitative robotic devices could provide more prolonged, more intense, and controlled periods of practice, often combined with other strategies that suit individuals’ needs (Hogan et al., 2006; Mazzoleni et al., 2017; Weber and Stein, 2018). Peripheral electrical stimulation, for instance, neuromuscular stimulation and functional electrical stimulation (FES), can also work on muscle atrophy, muscle tone, and motor neuron activation resulting in motor recovery in patients with paralysis after stroke (Sheffler and Chae, 2007; Knutson et al., 2015; Moon et al., 2017; Yang et al., 2019). The mu and beta rhythm suppression between active and passive conditions has been reported in lower limb rehabilitation. More suppression of mu and beta rhythm has been shown in active robot-assisted walking than in passive robot-assisted walking in cortical activation (Wagner et al., 2012). Robots allow control of individual and well-defined joint kinematics for coordinated functional movements. On the other hand, FES has not reached fine control of complex coordinated movements, particularly with surface electrodes (Koutsou et al., 2016; Shin and Hu, 2018). However, only FES can produce muscle contraction mimicking a natural voluntary contraction-induced movement because the agonist muscles contract, and the antagonist muscles are stretched during the passive movement. Therefore, robots combined with FES should lead to higher cortical excitation than executing the identical passive movement without concomitant FES. Furthermore, this integrated tool could recruit more receptors derived from muscle contractions in addition to passive proprioception, activating the reticular system more, which could produce more sensorimotor neural network excitation, and thus result in an interesting tool to provide neurofeedback in BMI-based motor rehabilitation paradigms. The brain somatosensory mu and beta ERD/ERS response during passive movement induced by FES and robot movements independently has been investigated (Crone et al., 1998; Müller et al., 2003; Müller-putz et al., 2007; Taylor, 2008; Cho et al., 2011; Shaikhouni et al., 2013; Vidaurre et al., 2016, 2019b,^2021^; Tu-Chan et al., 2017; Corbet et al., 2018; Hishinuma et al., 2019). However, when combining FES and robotic actuators to produce a sequence of passive movement, brain oscillatory response needs further investigation before being combined in a rehabilitation system.

Somatosensory cortical activation is essential in motor learning and rehabilitation, and multiple studies reported that sensory inputs affect the plasticity of sensorimotor systems in healthy humans and patients with brain injuries (Sanes and Donoghue, 2000; Rosenkranz and Rothwell, 2006; Edwards et al., 2019). However, the afferent contribution has not drawn attention as much as the efferent information during sensorimotor integration. As discussed, the mu and beta ERD/ERS (or suppression/enhancement) is neural correlates of cortical excitability supported by BOLD, fMRI, gamma EEG, and neuronal spikes. Therefore, it would be necessary to know how the cortex responds to somatosensory stimulation according to various stimulus patterns and types, which will provide cortical processing in response to different stimulation and valuable information in the design of neurofeedback devices to optimize afferent information in sensorimotor integration. In the present study, we hypothesized:(1) SMR in response to proprioceptive stimulation is suppressed/enhanced according to kinematic conditions and (2) more SMR suppression is observed in the somatosensory cortex in passive mechanical movement + FES than passive mechanical movement alone. For the kinematic aspect, the finger extension was divided into two phases: (1) dynamic extension and (2) static extension (holding a position). For the kinetics aspect, afferent brain activity (no simultaneous volition) was compared under two conditions of finger extension: (1) generated by a robotic hand orthosis and (2) generated by the orthosis simultaneously combined and assisted with FES.

## 2. Materials and methods

### 2.1. Experimental design

**FIGURE 1:**
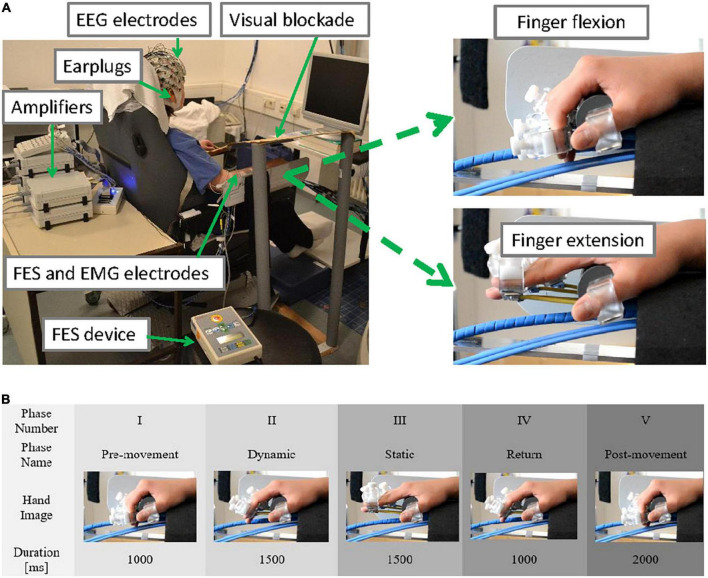
**(A)** Experimental setup (upper panel). The participant was sitting in a comfortable chair with the EEG cap on, the EMG electrodes placed over the forearm and upper arm muscles while the right hand was strapped to a robotic hand orthosis, and a pair of FES electrodes were attached to forearm muscles (left). A visual blockade (to prevent the user from receiving visual feedback from her right-hand movement) and earplugs were used. The maximal range of passive finger flexion (top right) and finger extension (bottom right) are shown. **(B)** Time course of one trial (lower panel). No stimulus was provided during the pre-movement period (phase I at –1,000 ms). No cue was presented. The fingers were extended according to the experimental conditions (ORTHOSIS or ORTHOFES) to reach a full finger extension (phase II at 0 ms). During the finger stretched period, the finger position was maintained (phase III at 1,500 ms). During the return period (phase IV at 3,000 ms), the fingers were returned to the starting position by the orthosis alone in both conditions (ORTHOSIS and ORTHOFES). No stimulus was delivered during the Post-movement period (phase V at 4,000 ms).

Ten healthy female volunteers aged between 22 and 38 years (nine right-handed, one left-handed) participated in the study. They were sitting upright in a comfortable chair facing a computer screen located 1 meter from the chair (Figure 1A). They were instructed to keep their gaze in the middle of the black computer screen and to remain still and relaxed during the measurement. A plastic panel was placed above the wrist as a visual blockade to prevent participants from seeing their hands being passively moved, and earplugs were used to prevent participants from hearing the different noises produced by using only the robot or the robot and FES combined. The two experimental conditions (robot only or ORTHOSIS and robot and FES combined or ORTHOFES) were randomly applied to their right hand in addition to one rest condition (REST) that was used as a reference or control.

In the robot condition (ORTHOSIS), each finger was extended individually using a custom-made hand orthosis using 4 DC-Motors M-28 (Kaehlig Antriebstechnik GmbH, Hannover, Germany) with a worm gearhead for each finger. The motor drove a Bowden cable via cogwheel and cog rail. A finger holder was mounted on the other side of each Bowden cable (details on the robotic orthosis’ construction can be found in Ramos-Murguialday et al. (2012, 2013). The maximum range of finger extension was determined at a position each participant felt comfortable. During the measurements (see Figure 1B), the participant’s fingers were extended by the orthosis to their predefined position (dynamic phase II) and maintained (static phase III). Then, the fingers were flexed automatically by the orthosis for all participants returning to the start position for the subsequent trial in the return phase IV.

In the orthosis and FES condition (ORTHOFES), two FES unipolar electrodes were placed over the extensor digitorum communis (EDC) muscle for the finger extension following anatomical landmarks. Before the measurement, the FES (UNAFET 8, UNA Systems, Belgrade, Serbia) parameters were configured for a finger extension. We adjusted the stimulation intensity with fixed 30 Hz and 300 μs pulses until fingers were extended at the same speed and in the same range of motion in ORTHOSIS. Both orthosis and FES concurrently induced the finger extension. The kinematics of the finger extension was identical to ORTHOSIS because the fingers were always in finger holders of the orthosis, limiting the movement (see Figure 1B). The fingers were extended by the orthosis and FES together after onset (dynamic phase II for 1.5 s) and were maintained in an extended position during the static period (phase III for 1.5 s). While fingers were being flexed in return phase IV for 1 s, FES was off, and the movement was produced by the orthosis alone (same as in the ORTHOSIS) for the subsequent trial.

No cue and random inter-trial intervals between 5 and 9 s were presented to minimize the subject’s anticipatory activity and allow the central nervous system (CNS) to return to baseline levels. The whole right arm was fixed to and positioned in an apparatus, which guaranteed negligible kinematic differences between conditions. Each condition was repeated 70 times. This study was approved by the ethics committee of the University of Tübingen, Medical Faculty.

### 2.2. Signal acquisition and processing

The EEG data were acquired using a BrainAmp (Brainproducts GmbH, Munich, Germany) with a sampling rate of 1,000 Hz. 64 EEG electrodes were referenced to the nasion and grounded anteriorly to Fz. The data were first filtered using a band-pass filter (2–45 Hz). Then, an independent component analysis-based method was used to detect and eliminate eye blinks and movement artifacts, and neck, cranial and facial EMG-related artifacts using Fieldtrip (Oostenveld et al., 2011). Afterward, data were spatially filtered using a short Laplacian (McFarland et al., 1997). In order to control for undesired peripheral muscle activity that could introduce confounds in the EEG processing, we recorded EMG in the moved and non-moved limbs. EMG data were collected with four bipolar Ag/AgCl electrodes and placed on muscles on both arms; one close to the external epicondyle on the extensor digitorum (forearm extension), the other on the flexor carpi radialis (forearm flexion), further on the external head of the biceps (upper arm flexion) and the last one placed on the external head of the triceps (upper arm extension). Then, they were processed using a high-pass filter at 10 Hz to detect unwanted upper limb movements. A trial was rejected and marked as “EMG contaminated” when it contained significant EMG activity irrelevant to the experimental design: any muscle activity in REST or ORHTOSIS; on the left arm and the right upper arm in ORTHOFES. In addition, trials were rejected if EMG activity was higher than three standard deviations (SD) from the baseline mean longer than 200 ms following (Ramos-Murguialday et al., 2012, 2013).

**FIGURE 2:**
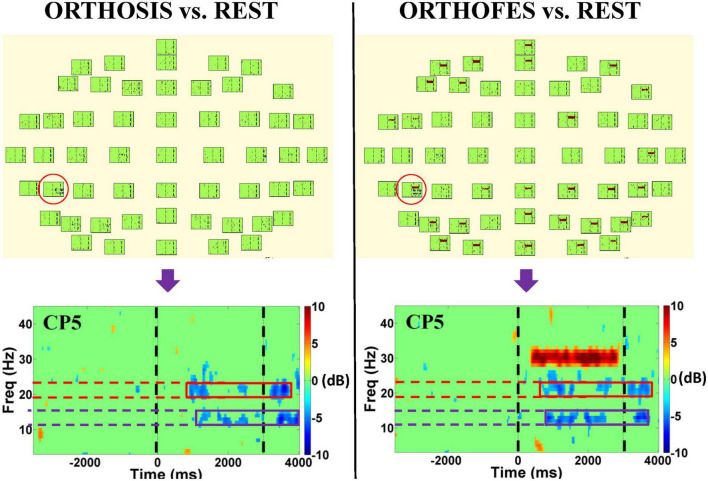
EEG spectral power bootstrap analysis during passive right-hand movement in one representative participant. The left and right panels show the differences in the time-frequency domain when ORTHOSIS and ORTHOFES conditions were compared to REST, respectively. CP5 was selected as a representative electrode (red circle) after visual inspection due to its more salient power suppression compared to other electrodes. The figures at the bottom show the enlarged plot of the selected electrode. The purple and red boxes indicate the frequency bands in which we found more significant power changes when using the bootstrap analysis. The green areas indicate no significant power differences compared to REST conditions. The color represents the power suppression, power enhancement, or no differences compared to the REST condition: blue being power suppression in dB, red being power enhancement in dB, and green being not significantly different (*P* < 0.01) from REST. The two black dashed vertical lines indicate the beginning (0 ms) and end of the passive movement during a trial (3,000 ms). The high spectral power in the range of 30 Hz was observed in the right panel during the trial period due to FES (stimulation frequency: 30 Hz) artifact, which was not found in ORTHOSIS (left panel).

After preprocessing, the spectral power of each EEG channel in experimental conditions (ORTHOSIS and ORTHOFES) was analyzed to detect the significance of within-subject and within-conditions (see Figure 2 and Supplementary material). The event-related spectral perturbation was then calculated using Morlet transforms (Daubechies, 1996) with 3 cycles at the lowest frequencies, 23.04 at the highest frequencies, a time window of 1,114 ms, and a 30 ms overlap. A 300 ms time window from −350 to −50 ms before the passive movement onset as a baseline for event-related spectra perturbation analysis. Forty-three linear-spaced frequencies were produced from 3 to 45 Hz and 200-time points. The brain activity in the experimental conditions was compared to the REST condition in a pairwise manner in the time-frequency domain to mask out the non-experimental as baseline condition: (ORTHOSIS vs. REST) and (ORTHOFES vs. REST). The spectral differences from REST were estimated using the EEGLAB toolbox.^1^ A null hypothesis distribution (*p* = 0.01) was calculated by accumulating surrogate data (200 bootstrap replications), shuffling the single-trial spectral DIFF estimates using a two-tailed bootstrap significance probability level implemented in the EEGLAB bootstrap method (Sivaganesan, 1994; Burgess and Gruzelier, 1999). Both sides of the surrogate distribution obtained for every frequency and time bin from the spectral DIFF were used for significance tests. Although we know this method is not corrected for multiple comparisons, we used it to orient further analysis reducing dimensionality in frequency band and electrodes (Ramos-Murguialday and Birbaumer, 2015). We observed significant band power changes in each subject when comparing ORTHOFES and ORTHOSIS to REST (see Supplementary material for more details).

EEG subject-specific frequency bands were visually selected based on the bootstrap analysis of each participant, as most statistically differential changes with a total significant period is longer than 600 ms between two conditions were detected in the time-frequency domain from movement-related afferent signals. Therefore, the manual selection of the frequency band was used to observe the profile of power reduction/enhancement over the entire trial rather than instantaneous power changes. Next, the preprocessed EEG signals were band-pass filtered according to these individual frequency bands and then squared. Finally, the proportional power decrease or increase to the activity during a baseline period (from −350 to −50 ms) at each electrode was averaged according to:


(1) $$Power\left[\%\right]=\frac{\left(E\,x\,p\,e\,r\,i\,m\,e\,n\,t\,a\,l-B\,a\,s\,e\,l\,i\,n\,e\right)}{B\,a\,s\,e\,l\,i\,n\,e}\times 100$$

with *Experimental* and *Baseline* in Equation 1 denoting the activity of each electrode during the experimental condition (ORTHOSIS and ORTHOFES) and baseline period, respectively.

The relative power calculated from Equation 1 was used to compare power dynamics differences between ORTHOSIS and ORTHOFES. A two-sided Monte-Carlo permutation test at the 5% significance level was applied to the mean band power of all the participants’ trial-based data to test the hypothesis that the band power decrease (ERD) during ORTHOFES is significantly different compared to the one during ORTHOSIS. We used 1,000 repetitions of Monte-Carlo procedures to find the time points with the significant mean difference between two conditions, and *p*-values were corrected by the False Discovery Rate (FDR) described by Benjamini and Hochberg (1995) for multiple comparisons in time series. We also analyzed the power of the sensorimotor frequency band at electrode C3, which is commonly used to study motor-related EEG oscillations during upper limb movement. Even though each subject may have slightly different frequency bands for motor commands (mu rhythm ERD) in neurofeedback tasks, we analyzed 8–13 Hz to observe the influence of the induced movement on EEG oscillations over the electrode C3.

## 3. Results

The data from two participants were not included in the group analysis due to no changes in the entire frequency band (*n* = 1) and the noisy EEG (*n* = 1), of which the spectral power maps are seen in Supplementary Figures 18, 19. After conservative trial rejections based on EMG and EEG artifacts, the number of clean trials available for REST, ORTHOSIS, and ORTHOFES across participants were 35.4 ± 9.6 (mean ± SD), 36.6 ± 9.1, and 39.0 ± 9.0 respectively, being EMG artifacts the primary source of trial elimination in line with previous work (Ramos-Murguialday et al., 2013; Ramos-Murguialday and Birbaumer, 2015; López-Larraz et al., 2018; Ray et al., 2020).

**FIGURE 3:**
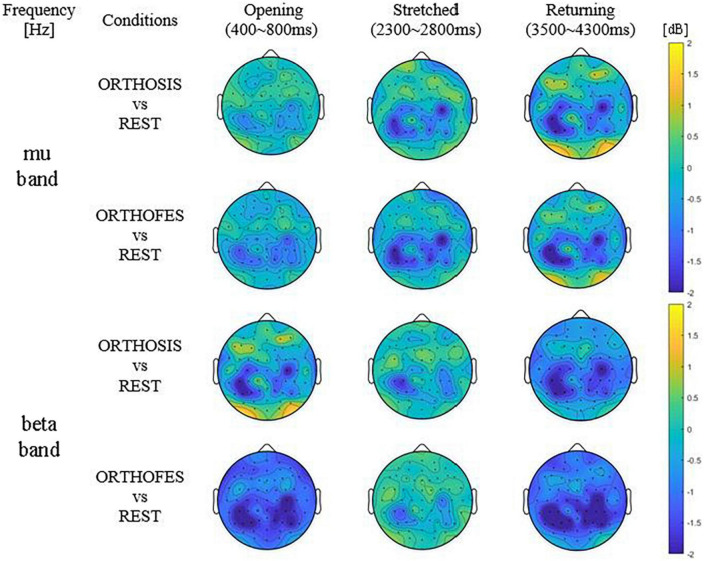
Averaged differential power values of all included participants’ data. Topography presenting the power changes in ORTHOSIS and ORTHOFES averaged (*N* = 8). The frequency band of each subject was selected from the above information (Table 1). The power changes in ORTHOSIS and ORTHOFES were compared to REST. The pronounced power reduction was observed in contralateral S1 and ipsilateral M1 and S1—the color bars fixed scale (–2 to 2 dB) for comparisons between conditions and phases. (S1 = primary somatosensory cortex, M1 = primary motor cortex). Yellow indicates ERS (power increase in dB), and blue shows ERD (power decrease in dB) compared with REST, as expressed in the color bar.

**FIGURE 4:**
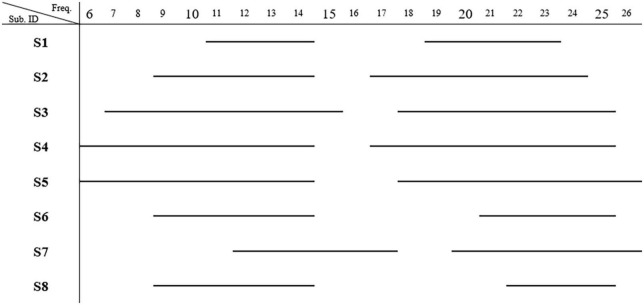
Most representative frequency bands. The frequencies present significant band power changes in each subject. Two frequency bands showing significant power changes (*p* < 0.01) were observed (*N* = 8) after bootstrap analysis. The mu band was 11.3 ± 2.6 (mean ± SD) Hz, and the beta band was 21.8 ± 2.4 Hz.

**FIGURE 5:**
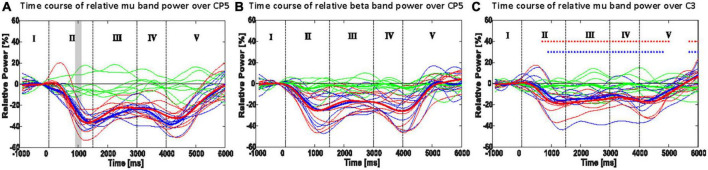
Time course of the most responsive electrode in power (ERD/ERS): CP5 or P5 of mu rhythm **(A)** and beta rhythm **(B)**; C3 in mu (8–13 Hz) power **(C)**. Thin lines show the mean band power of a single participant, and thick lines indicate the mean of the thin lines. Shaded areas in panels **(A,B)** show the 100 ms time bins, which resulted in a significant difference between the ORTHOFES and ORTHOSIS conditions using a two-sided Monte Carlo permutation test (α = 0.05). Red asterisks (*) in panel **(C)** indicate the 100 ms time bin, which has a significant difference between REST and ORTHOFES of grand averages (thick lines) with a two-sided Monte-Carlo permutation test (α = 0.05). Blue asterisk (*) indicates the same between REST and Orthosis. Green, red, and blue lines represent REST, ORTHOFES, and ORTHOSIS, respectively. I: pre-movement phase, II: opening phase, III: stretched phase, IV: returning phase, and V: post-movement phase.

CP5 in seven subjects and P5 in one subject presented the most salient expected power changes in the contralateral hemisphere (see Figures 2, 3). Two common discrete frequency bands typically analyzed were individualized and used for further analyses: the mu (11.3 ± 2.6 Hz) and the beta (21.8 ± 2.4 Hz), as shown in Figure 4. The significant differences between ORTHOSIS and ORTHOFES were observed in time windows during movement in the mu (see Figure 5A). In the dynamic period (phase II, 900–1,100 ms), ORTHOFES produced a faster mu suppression, which induces significantly more cortical excitation (i.e., more ERD) than ORTHOSIS during this specific time window. However, both conditions reach similar maximum levels of mu suppression. In the static period (phase III, 2,000–2,400 ms), the movement stopped after reaching the predefined range of motion. In beta rhythm, no other significant differences were observed between ORTHOFES and ORTHOSIS.

**TABLE 1:** Most representative electrodes per participant.

Sub. ID	Contralateral (left) hemisphere						Ipsilateral (right) hemisphere					
S1				**CP5**								
S2				**CP5**								
S3				**CP5**			C4		CP2	CP4		P2
S4		C3		**CP5**		P5	C4				CP6	
S5			CP3	**CP5**		P5	C4			CP4		
S6	C1			CP5	P3	**P5**	C4	C6				
S7	C1	C3		**CP5**	P3		C4					P2
S8				**CP5**								

Electrodes of the significant (bootstrap analysis) band power changes compared to REST in ORTHOSIS and ORTHOFES during passive movement of the right hand. The band power decrease (or ERD) occurred on both hemispheres in five subjects (S3, S4, S5, S6, and S7). The most responsive electrode of the contralateral hemisphere was CP5 (*N* = 7) and P5 (*N* = 1), as indicated in bold.

The ERD/ERS changes of mu rhythm (8–13 Hz) are shown in Figure 5C for the right-hand movement. When each condition was compared to REST, both conditions presented significantly larger cortical excitation (larger ERD) since the pre-movement phase (baseline). In phase V, we observed a significant difference due to the time needed for the oscillatory brain activity to return to baseline.

## 4. Discussion

Using a bootstrap analysis to identify the most salient power changes allowed us to determine the most representative electrode over the sensorimotor cortex to focus our analysis. Sensors CP5 or P5 of the contralateral hemisphere presented the most salient power changes in mu and beta bands when passively extending and flexing the fingers using a robotic orthosis alone or combined with FES. Focusing on these representative electrodes allowed us to reduce the problem’s dimensionality and run a subsequent permutation analysis to compare ERD/ERS changes during the movement sequence between conditions on mu and beta bands independently. The suppression of visual and auditory stimuli, the cue presentation protocol, and the control of the movement trajectory (range of motion) left haptic and proprioceptive receptors as the only movement-related generators of afferent activity we could measure with EEG. Our results describe the evolution of the somatosensory mu rhythm recorded with EEG during a finger extension/flexion passive movement sequence, i.e., moved by external devices with no subject’s intention. Furthermore, our experimental protocol allowed us to isolate the influence of muscle spindle recruitment in the sensory mu rhythm evolution during the movement sequence.

Our results align with previous literature and confirmed similar mu rhythm evolutions during passive-movement-related brain oscillatory activity (Pfurtscheller and Lopes da Silva, 1999; Alegre et al., 2004; Ramos-Murguialday and Birbaumer, 2015). In both mu and beta rhythms, ERD peaks (local minima in power) were produced only during or immediately after dynamic movement phases (phases II and V). During the movement sequence static period (phase III), the ERD was sustained but decreased continuously until the following dynamic condition (return period, phase IV) began. Even though the ERD decreased during the static condition of phase III, the power decrease or ERD was constantly significantly larger than during rest (baseline), suggesting cognitive processing sustains the ERD. We speculate that the observed cortical activity is produced because the brain processes the position as part of a passive movement sequence, similar to what has been found in active movement sequences (Alegre et al., 2002, 2004), which showed that ERD/ERS is related to the whole motor process, and not to each sub-movement or subsequence. However, as far as we know, our data demonstrate this effect for the first time during pure passive movement only. Our observation is reinforced by the incremental ERD decrease initiated during the static phase, which was very different from the ERD decrease observed in post-movement phase V (end of the movement sequence). The change in ERD at the end of the movement sequence was faster (shorter latency returning to baseline level or steeper return to baseline level). Another plausible explanation could be that the observed cortical activity is produced because the subjects were cognitively paying attention to their proprioceptive signals (visual and auditory feedback was blocked) or as a result of motor control theory, feedforward control based on sensory input forecasting (Sun et al., 2015; Branco et al., 2017).

Between 900 and 1,100 ms after stimuli onset during passive finger extension (phase II), we observed a significantly larger mu ERD with FES than without FES, reflecting a significantly faster ERD increase when FES and orthosis were combined to produce the passive movement. However, the maximum ERD (i.e., peak) was not significantly different between conditions. This expected result confirms that the induced passive movement via both electrical stimulation and orthosis stimulated more receptors, causing a faster but not a larger ERD on the sensory cortex. Based on previous work (Stancák et al., 2003; Insausti-Delgado et al., 2021), we expected a more prominent ERD peak for the ORTHOFES condition. Therefore, the combination of different receptors might not increase the amplitude but the modulation speed of the ERD. Our experimental design isolated artificial electrically induced muscle contraction as the only difference between conditions. The firing rate of GTOs is muscle-force-dependent (Paillard and Brouchon, 1968; Purves et al., 2004), and that of the muscle spindles depends on muscle length or velocity (Chye et al., 2010). Thus the effect may come from a more considerable afferent inflow to cortical structures due to the extra firing of skin mechanoreceptors, mainly GTO, and muscle spindles excited by the FES-induced muscle contraction.

Another explanation of the faster but more considerable decrease in mu-band power during ORTHOFES, albeit unlikely, could be due to greater cognitive attention during FES than during orthosis during the first second after onset. Multiple studies reported that attention modulated somatosensory mu rhythm (Babiloni et al., 2004, 2008; Jones et al., 2010; Anderson and Ding, 2011; Wiesman and Wilson, 2020). None of the participants in the present study had experienced electrical stimulation in their bodies before. Therefore, the FES-related arousal might have occurred only at the beginning of stimulation, the dynamic phase, or the experiment. Still, we did not see any difference between trials at the beginning and end of the experimental session in the ERD that could explain this option.

Interestingly, during the static condition (phase III), the mu ERD decrease was different in both conditions in the mu band only, contradicting previous work indicating that EEG beta activity is related to the afferent activity (Alegre et al., 2002). Our results demonstrate that changes in afferent activity are reflected mainly in the mu band and primarily in the ERD’s modulation speed. The ERD decreased at the beginning of the static phase. In both conditions, there was no movement and no change in receptors excitation compared to the previous movement sequence phase. In the ORTHOFES condition, the extensor muscle stimulation induced an isometric passive contraction, which could produce more afferent stimulation than in the ORTHOSIS condition (equivalent to resting in terms of movement). Indeed, the ORTHOFES condition during the static phase could be compared to a muscle contraction against resistance, which usually induces extra inhibition of antagonists’ muscles when the movement is actively performed (Katz et al., 1991; Perlmutter et al., 1998). Intuitively, one would assume that if ERD is produced by sensory receptors excitation during passive movement, the ORTHOFES condition should present a larger ERD or at least a slower decrease of ERD, but this was not the case.

As opposed to the conventional understanding of contralateral S1 activation in sensory stimulation, the electrode over M1 in the ipsilateral hemisphere showed a significant mu ERD in response to the proprioceptive information caused by the passive movement. The role of the ipsilateral M1 activation has not been elucidated yet. However, this result agrees with the previous study, which investigated the interferences of afferent feedback in the mu rhythm modulation (Hommelsen et al., 2017). Furthermore, a recent motor task study showed that ipsilateral M1 ERD was related to fine hand and finger movements and the ability to maintain a steady level of contraction (Porcaro et al., 2021). Therefore, the ERD in ipsilateral M1 might have been caused to maintain a steady hand and finger posture during the trials in the present study.

In the neurofeedback system based on mu rhythm, subjects’ movement intention is usually classified against rest (or idle state) in the absence of any sensorimotor feedback (except for visual or auditory feedback (Birbaumer et al., 1999; Sellers and Donchin, 2006; Takahashi et al., 2008; Furdea et al., 2009; Ramos-Murguialday et al., 2012) or the presence of vibrotactile or proprioceptive stimuli (Pfurtscheller et al., 1998; Klimesch, 1999; Cassim et al., 2000; Chatterjee et al., 2007; Ramos et al., 2009). One of the challenges in asynchronous feedback devices (Ramos-Murguialday et al., 2012; Vidaurre et al., 2016) is the insufficient understanding of the effects of sensory information inflow from the brain-controlled peripheral stimulation (Cho et al., 2011; Hoffmann et al., 2011; Vidaurre et al., 2013). The previous study showed that the afferent information can cause false positives by the classifier in detecting motor commands, particularly in the presence of sensory information induced incorrectly or accidentally without motor intentions (Hommelsen et al., 2017). Therefore, the afferent feedback should be considered in the design of the closed-loop rehabilitation system, such as using spatial filters for only motor-related mu-rhythm. In addition, more attention should be paid to investigating the simultaneous efferent and afferent components for a contingent link between brain signals and feedback devices (e.g., exoskeleton or FES).

Even though no significant difference between the two conditions was found in the second dynamic period (phase IV), the suppression in mu rhythm induced during ORTHOSIS tends to be faster and larger compared to the ORTHOFES condition. This trend could reflect a net increase in afferent activity change in the cortex, as during ORTHOFES, muscle contraction occurred during the previous static phase. We demonstrate that the net increase in afference cortical activity is larger in the transition between posture and movement during ORTHOSIS than during the ORTHOFES condition. This trend indicates that the afference produced by the contribution of electrical stimulation (afference related to muscle contraction and mechanoreceptors) has a significantly lower influence on alpha ERD than the one evoked simply by proprioception. Therefore, the movement of the limb (the receptors related to it) is the principal component of the afferent activity. The trend has important implications for neurorehabilitation based on Brain-Machine-interfaces. It highlights proprioceptive feedback as probably the best option to induce cortical changes based on afferent signals to close the loop between the brain and movement. We are aware that the number of subjects studied in the here presented work is a limitation, but we compensated for this with our conservative artifact rejection and processing methods. However, further studies with a higher number of subjects are necessary to confirm this tendency.

It is known that beta ERS (or beta rebound) is induced after movement, reflecting the processing of sensorimotor information in the previous phase for an inhibitory rebound after excitation (Pfurtscheller et al., 1998; Cassim et al., 2000; Alegre et al., 2002). Different movements of the same limb could be classified by decoding EEG beta rebound in a post-movement period (Pfurtscheller et al., 1998). In our results, the post-movement beta-band rebound was not different between conditions in any of the bands analyzed, which might not reflect the difference in afferent information processing between conditions during the entire movement sequence but only during the last movement sub-sequence or phase, which was identical (phase V in Figure 5B). Unfortunately, our experimental protocol does not allow us to study the sensory post-processing difference between conditions. FES was applied during hand opening (phase II) and hand-stretched (phase III), but the hand was closed only by the orthosis in both ORTHOSIS and ORTHOFES conditions. During the closing phase, the afferent differences between conditions might have been washed out because it takes about a few seconds for the ERD to return to the baseline (Cassim et al., 2000).

## 5. Conclusion

Our results indicate that mu rhythm was influenced considerably by muscle kinematics during finger flexion/extension produced by external devices, which has relevant implications for the design of neuromodulation and neurorehabilitation. Besides, the ERD decrease during the static condition (as part of a movement sequence) represents cognitive processing sustaining the ERD (as there is no movement at all). As far as we know, this is the first time this effect has been demonstrated during pure passive movement only. Furthermore, we showed that the combination of different movement afferent receptors might not increase the amplitude, but the modulation speed of the ERD and that proprioception is the principal component of the afferent activity during the passive movement.

## Data availability statement

The original contributions presented in this study are included in the article/Supplementary material, further inquiries can be directed to the corresponding author.

## Ethics statement

The studies involving human participants were reviewed and approved by Prof. Dr. med. D. Luft, Ethik-Kommission an der Medizinischen Fakultät der Eberhard-Karls-Universität und am Universitätsklinikum Tübingen. Written informed consent for participation was not required for this study in accordance with the national legislation and the institutional requirements.

## Author contributions

WC contributed to the research design and implementation, data acquisition, analysis of the results, and the manuscript. AR-M supervised the whole process of data acquisition, analysis, and manuscript revision. NB managed the entire project and reviewed the manuscript. JA participated in the scientific input and contributed to the manuscript revision. CV aided in interpreting the results and revising the manuscript. All authors contributed to the article and approved the submitted version.

## Footnotes

1
http://sccn.ucsd.edu/eeglab/
