# Supplementary material for: Cortical processing during robot and functional electrical stimulation
Source: Front Syst Neurosci. 2023 Mar 21;17:1045396. doi: 10.3389/fnsys.2023.1045396 (PMC10070684; doi:10.3389/fnsys.2023.1045396)
Supplement: Supplementary file 1 [file Data_Sheet_1.DOCX]

Supplementary Material

# Selection of frequency bands in an EEG electrode


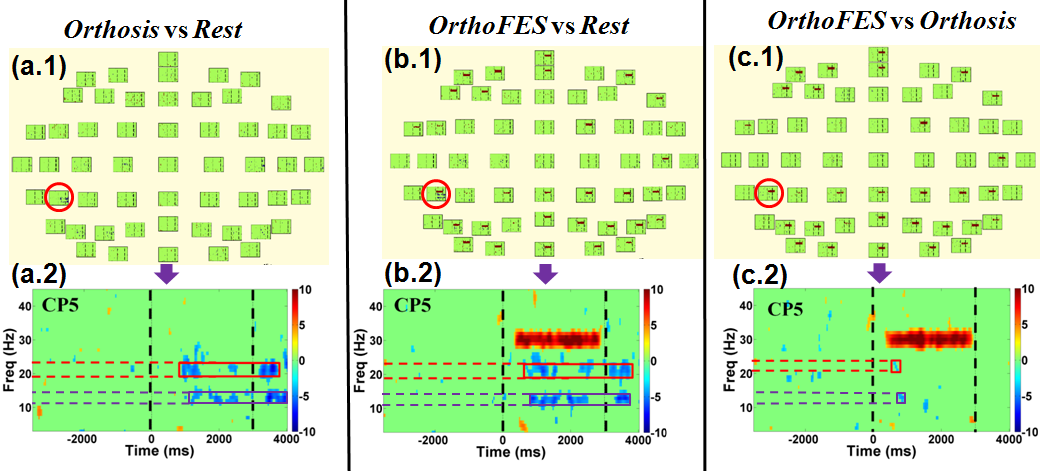


**Supplementary Figure 1.** EEG event related spectral power bootstrap analysis. (a.1) and (b.1) show EEG activity produced by right hand finger extension during Orthosis and OrthoFES conditions respectively compared with Rest condition. (c.1) shows the spectral power differences between OrthoFES and Orthosis. CP5 was selected as a representative electrode (red circle) due to its greater significant ERD after bootstrap analysis compared to other electrodes. Figure (a.2), (b.2), and (c.2) indicate the relevant significant frequency bands of the selected electrode, CP5, in each comparison. The purple and red boxes indicate the most significant frequency bands during Orthosis and OrthoFES conditions. The two black dashed vertical lines indicate the beginning (0 ms) and end of a movement during single trial (3000ms). The high spectral power in the range of 30 Hz were observed in the panel (b) and (c) during the trial period. FES (stimulation frequency: 30Hz) caused this artifact which was not found in Orthosis condition, panel (a).


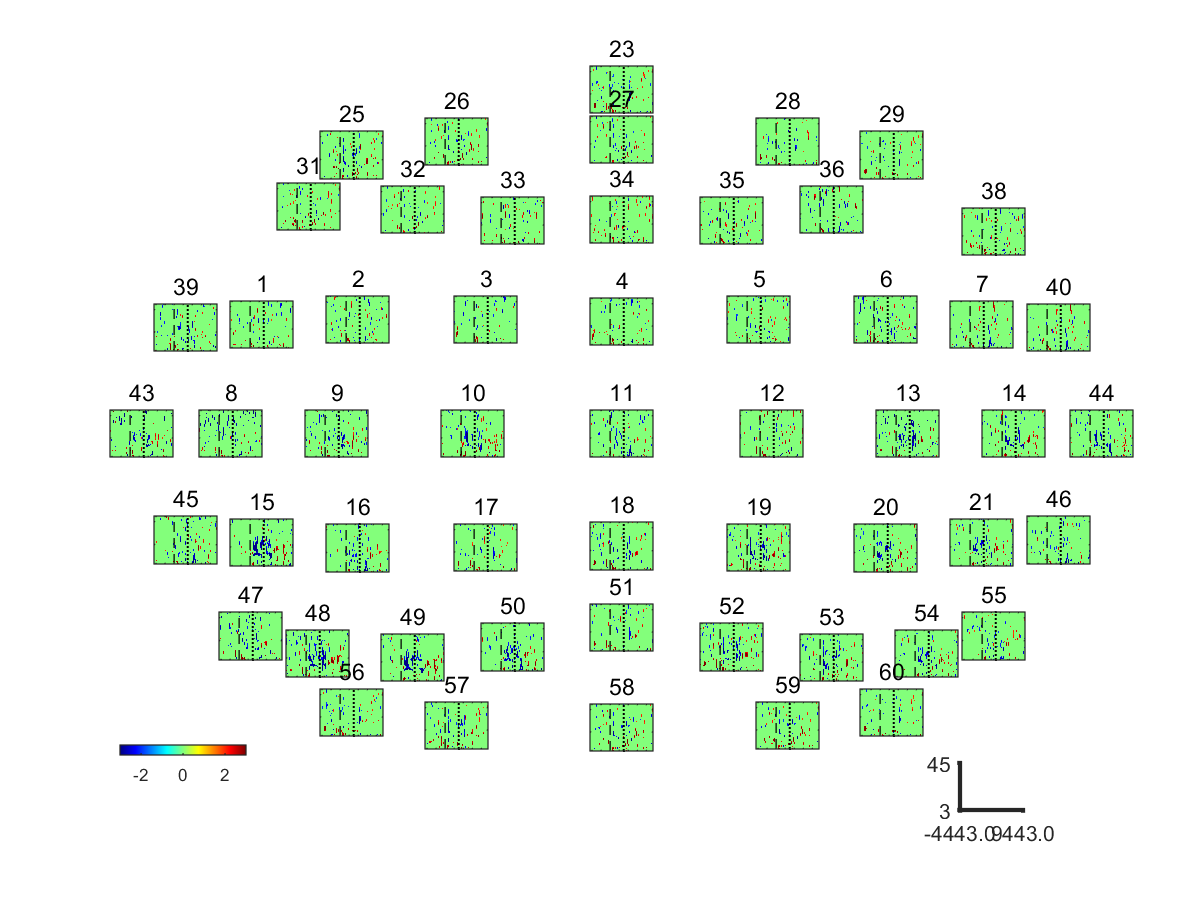


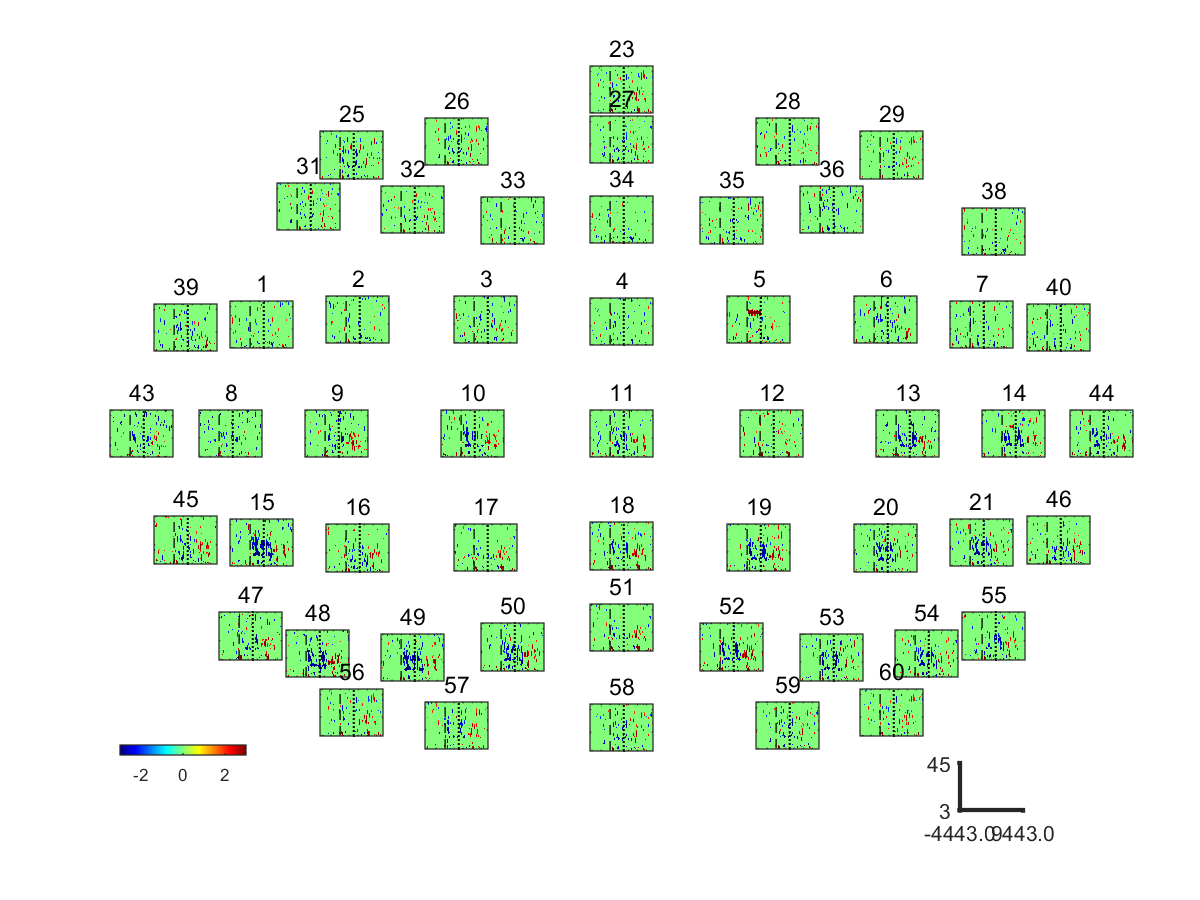


**Supplementary Figure 2.**. EEG event related spectral power bootstrap analysis of **S1**: ORTHOSIS vs. REST (Top) and ORTHOFES vs. REST (Bottom)

| Frequency | Conditions | Phase II and III | Phase IV and V |
| --- | --- | --- | --- |
| Alpha  Band | ORTHOSIS  Vs  REST | 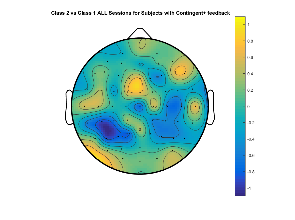 | 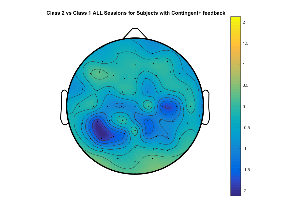 |
|  | ORTHOFES  Vs  REST | 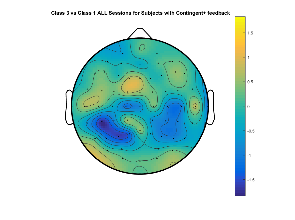 | 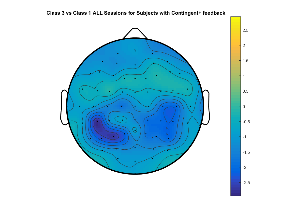 |
| Beta  band | ORTHOSIS  Vs  REST | 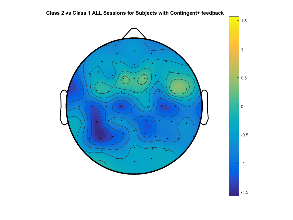 | 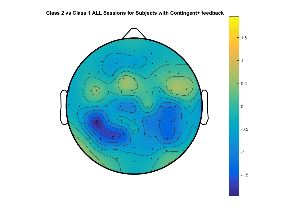 |
|  | ORTHOFES  Vs  REST | 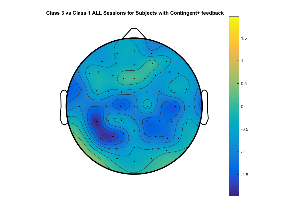 | 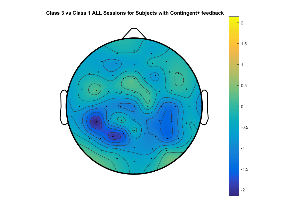 |

**Supplementary Figure 3.**. Averaged differential power values of **S1**. Topography presenting the power changes in ORTHOSIS and ORTHOFES. The 11 ~ 14 Hz and 19 ~ 23 Hz were selected for Alpha and Beta band respectively. The power changes in ORTHOSIS and ORTHOFES were compared to REST. The color bars were autoscaled to detect the meaningful area between conditions and electrodes. Yellow indicates ERS (power increase in dB), and blue shows ERD (power decrease in dB) compared with REST, as expressed in the color bar.


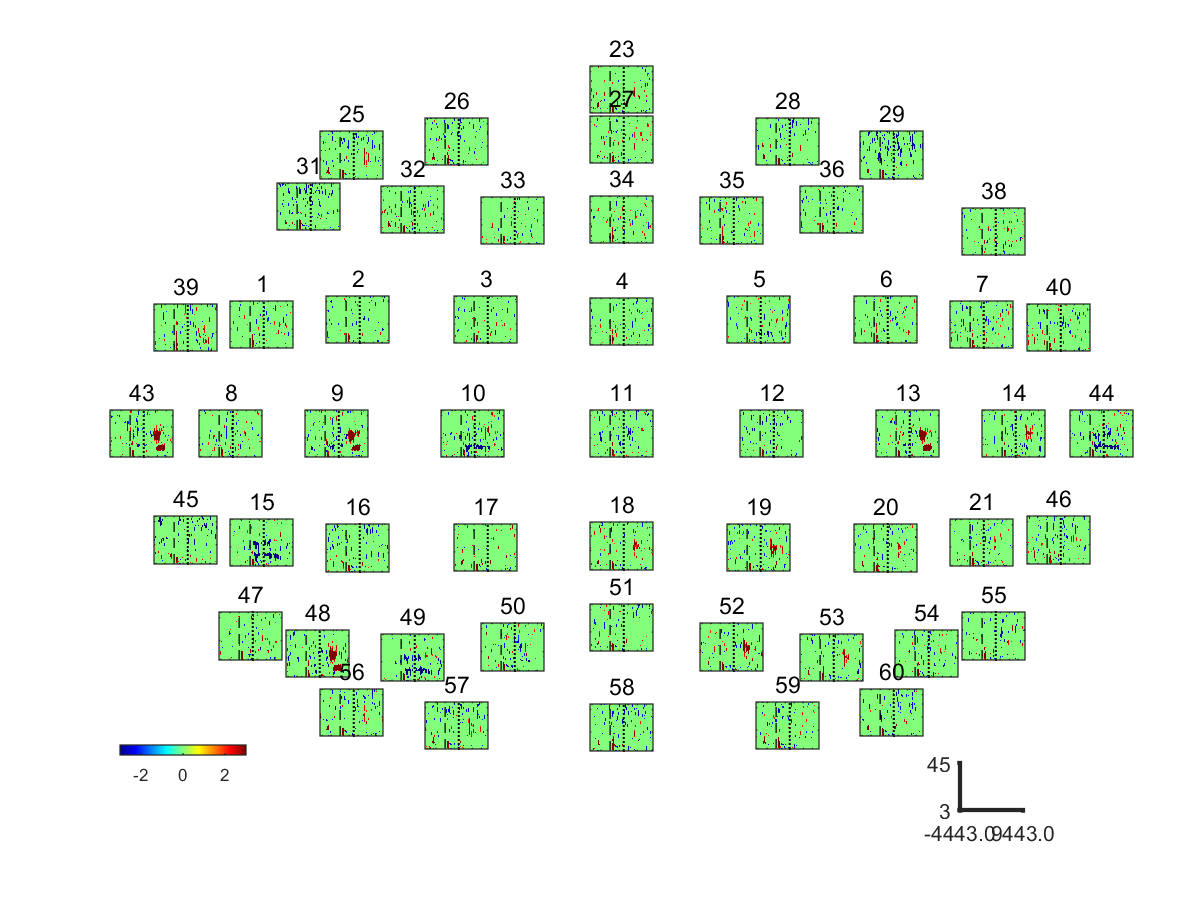

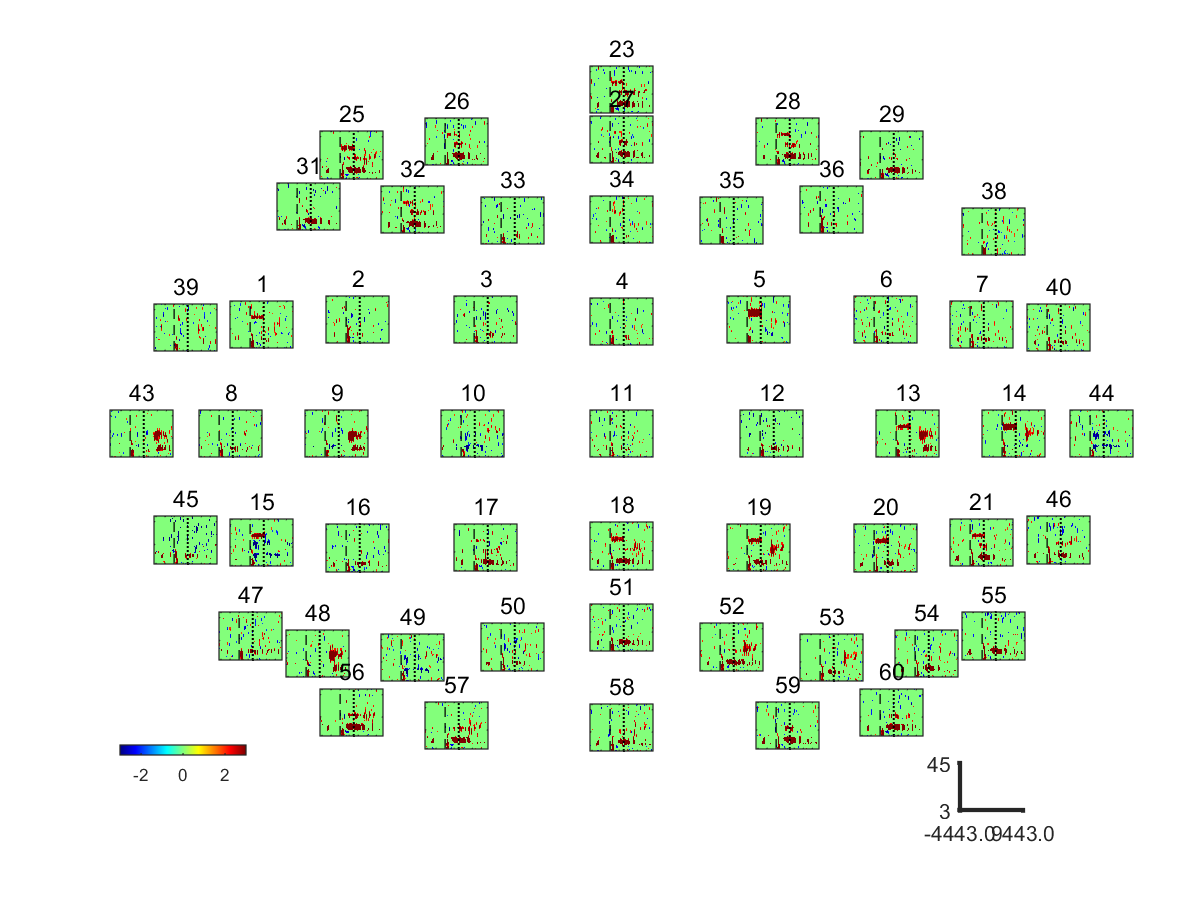


**Supplementary Figure 4.** EEG event related spectral power bootstrap analysis of **S2**: ORTHOSIS vs. REST (Top) and ORTHOFES vs. REST (Bottom)

| Frequency | Conditions | Phase II and III | Phase IV and V |
| --- | --- | --- | --- |
| Alpha  Band | ORTHOSIS  Vs  REST | 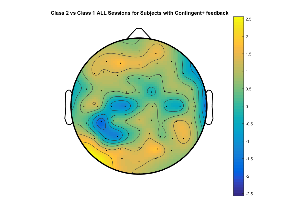 | 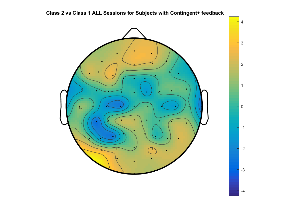 |
|  | ORTHOFES  Vs  REST | 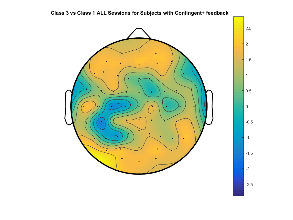 | 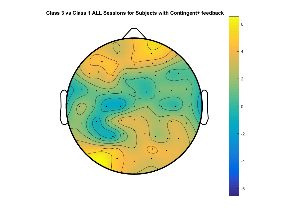 |
| Beta  band | ORTHOSIS  Vs  REST | 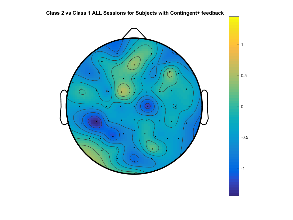 | 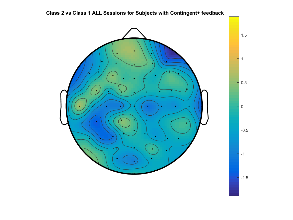 |
|  | ORTHOFES  Vs  REST | 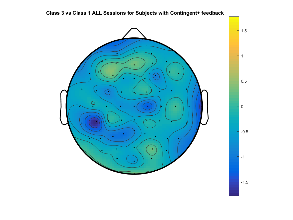 | 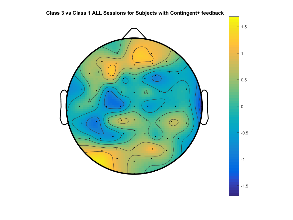 |

**Supplementary Figure 5.**. Averaged differential power values of **S2**. Topography presenting the power changes in ORTHOSIS and ORTHOFES. The 9 ~ 14 Hz and 17 ~ 24 Hz were selected for Alpha and Beta band respectively. The power changes in ORTHOSIS and ORTHOFES were compared to REST. The color bars were autoscaled to detect the meaningful area between conditions and electrodes. Yellow indicates ERS (power increase in dB), and blue shows ERD (power decrease in dB) compared with REST, as expressed in the color bar.


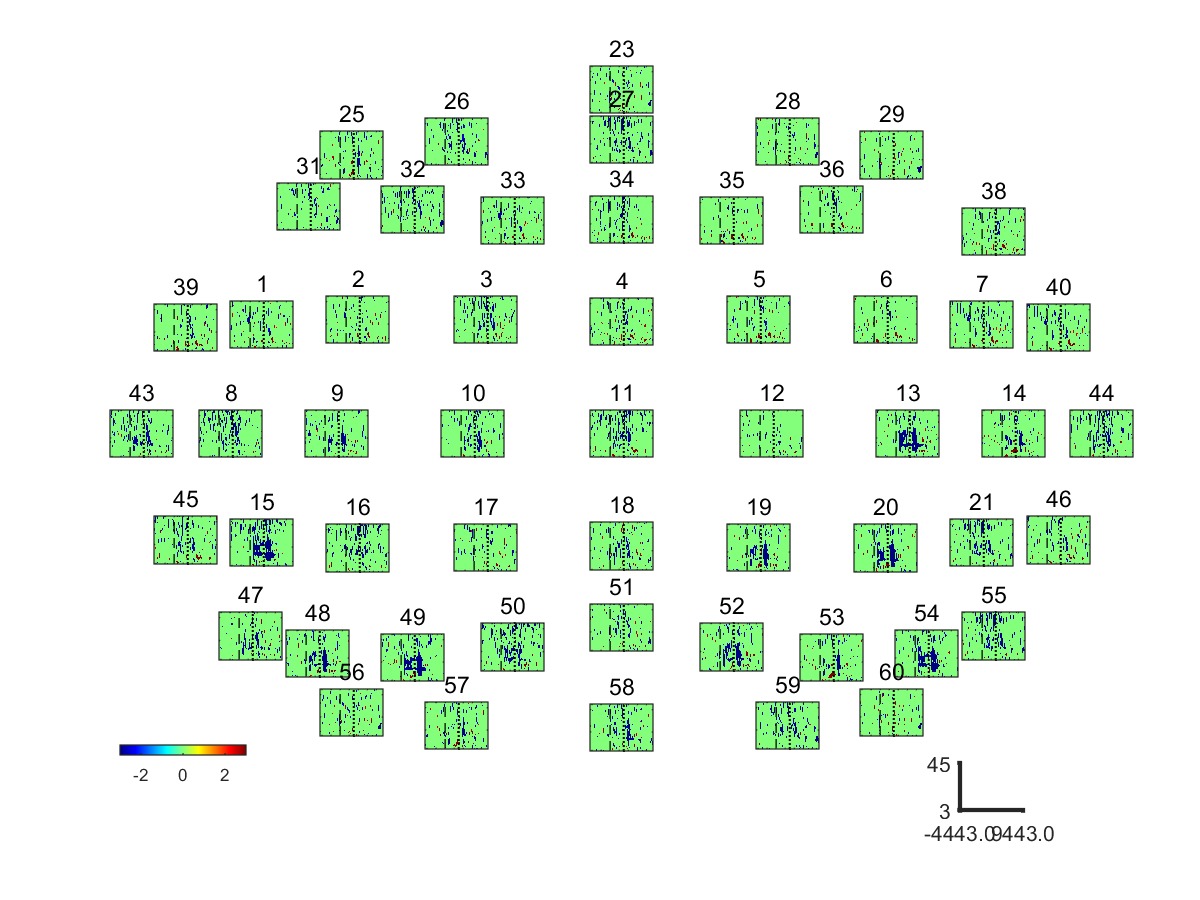

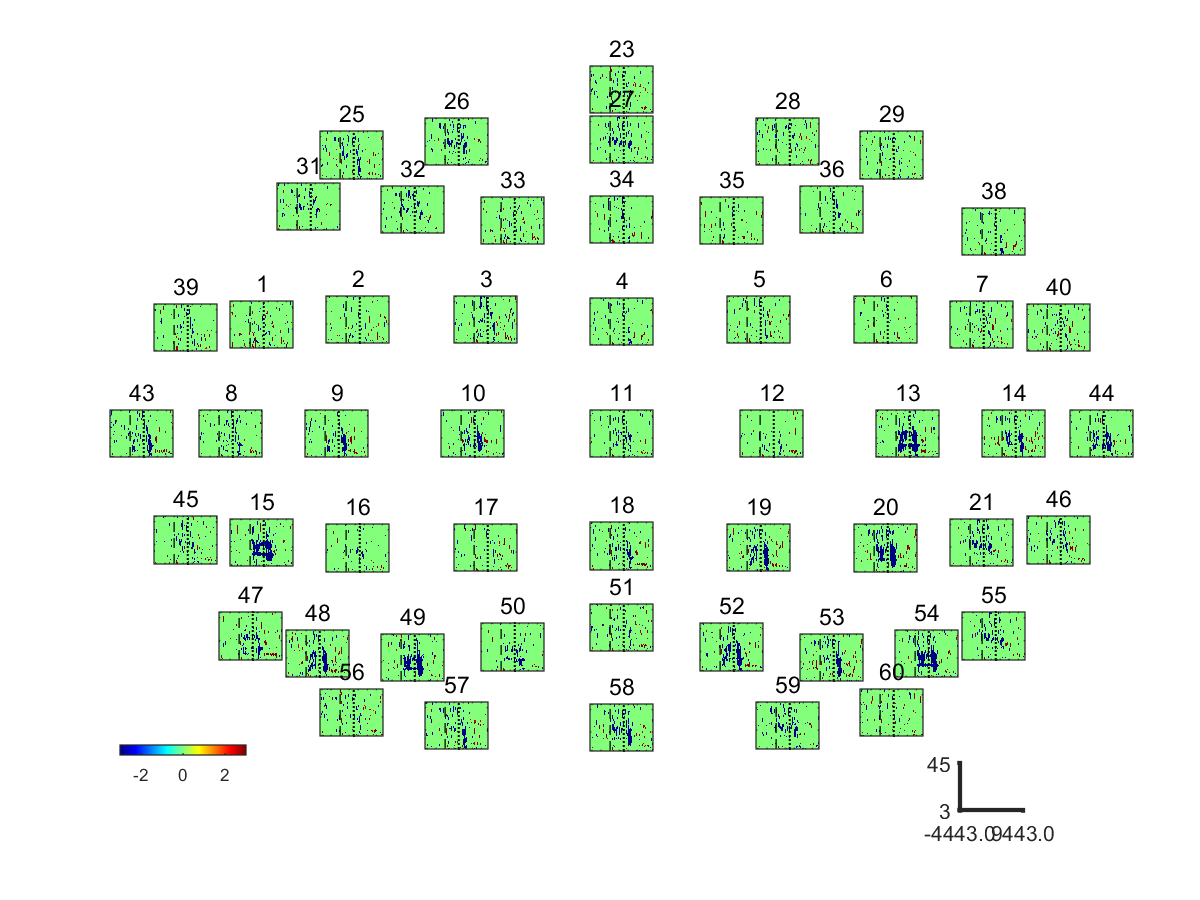


**Supplementary Figure 6.** EEG event related spectral power bootstrap analysis of **S3**: ORTHOSIS vs. REST (Top) and ORTHOFES vs. REST (Bottom)

| Frequency | Conditions | Phase II and III | Phase IV and V |
| --- | --- | --- | --- |
| Alpha  Band | ORTHOSIS  Vs  REST | 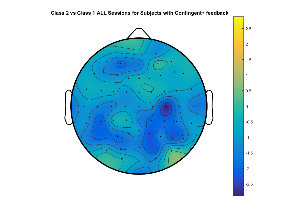 | 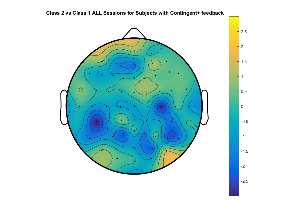 |
|  | ORTHOFES  Vs  REST | 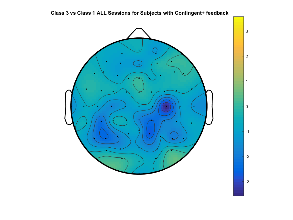 | 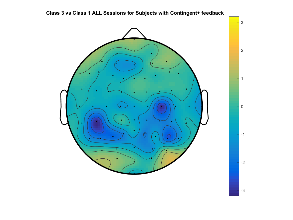 |
| Beta  band | ORTHOSIS  Vs  REST | 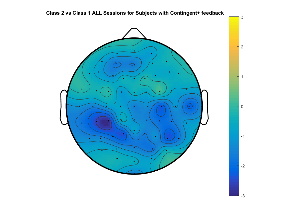 | 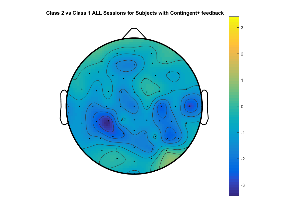 |
|  | ORTHOFES  Vs  REST | 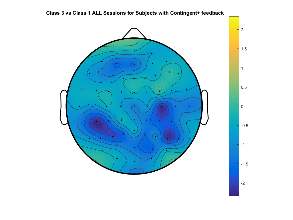 | 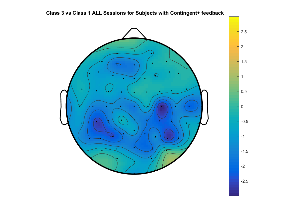 |

**Supplementary Figure 7.**. Averaged differential power values of **S3**. Topography presenting the power changes in ORTHOSIS and ORTHOFES. The 7 ~ 15 Hz and 18 ~ 25 Hz were selected for Alpha and Beta band respectively. The power changes in ORTHOSIS and ORTHOFES were compared to REST. The color bars were autoscaled to detect the meaningful area between conditions and electrodes. Yellow indicates ERS (power increase in dB), and blue shows ERD (power decrease in dB) compared with REST, as expressed in the color bar.


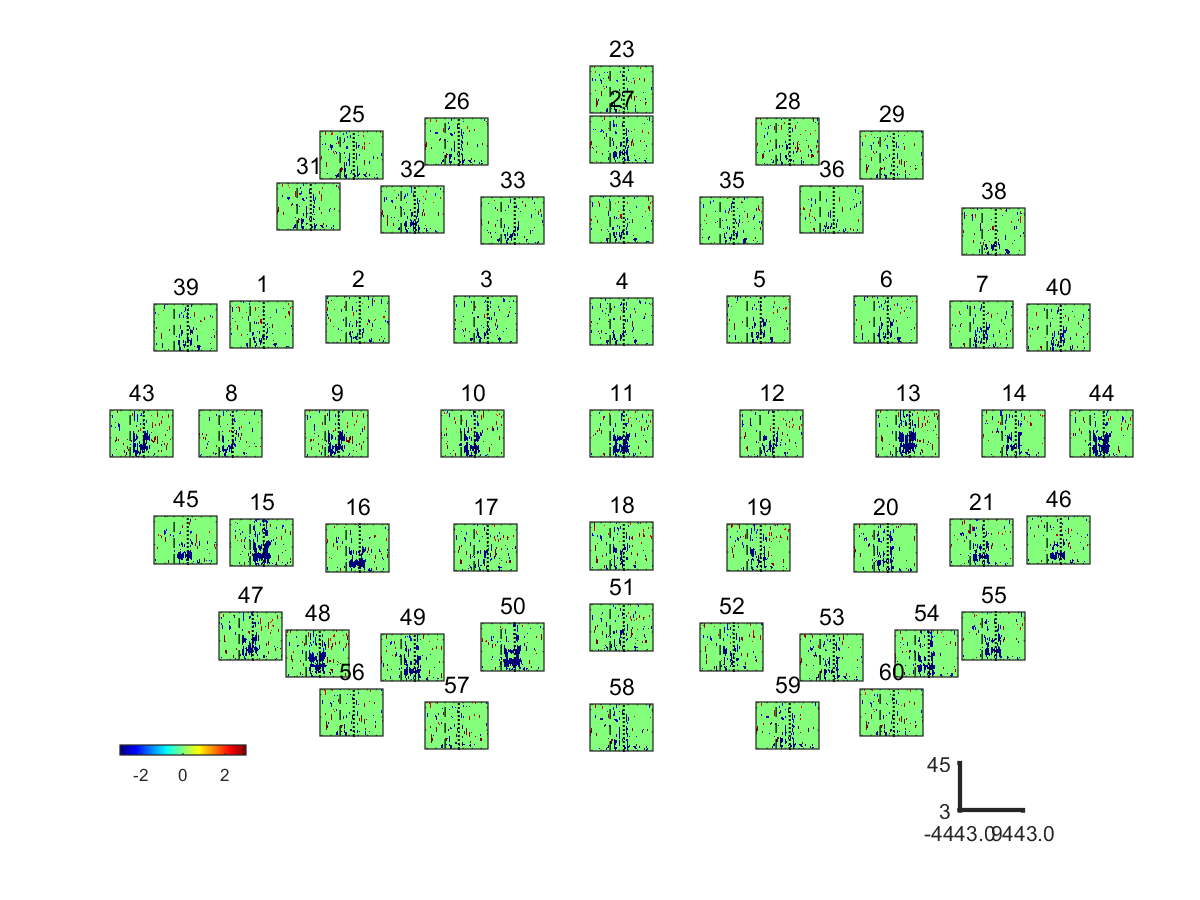

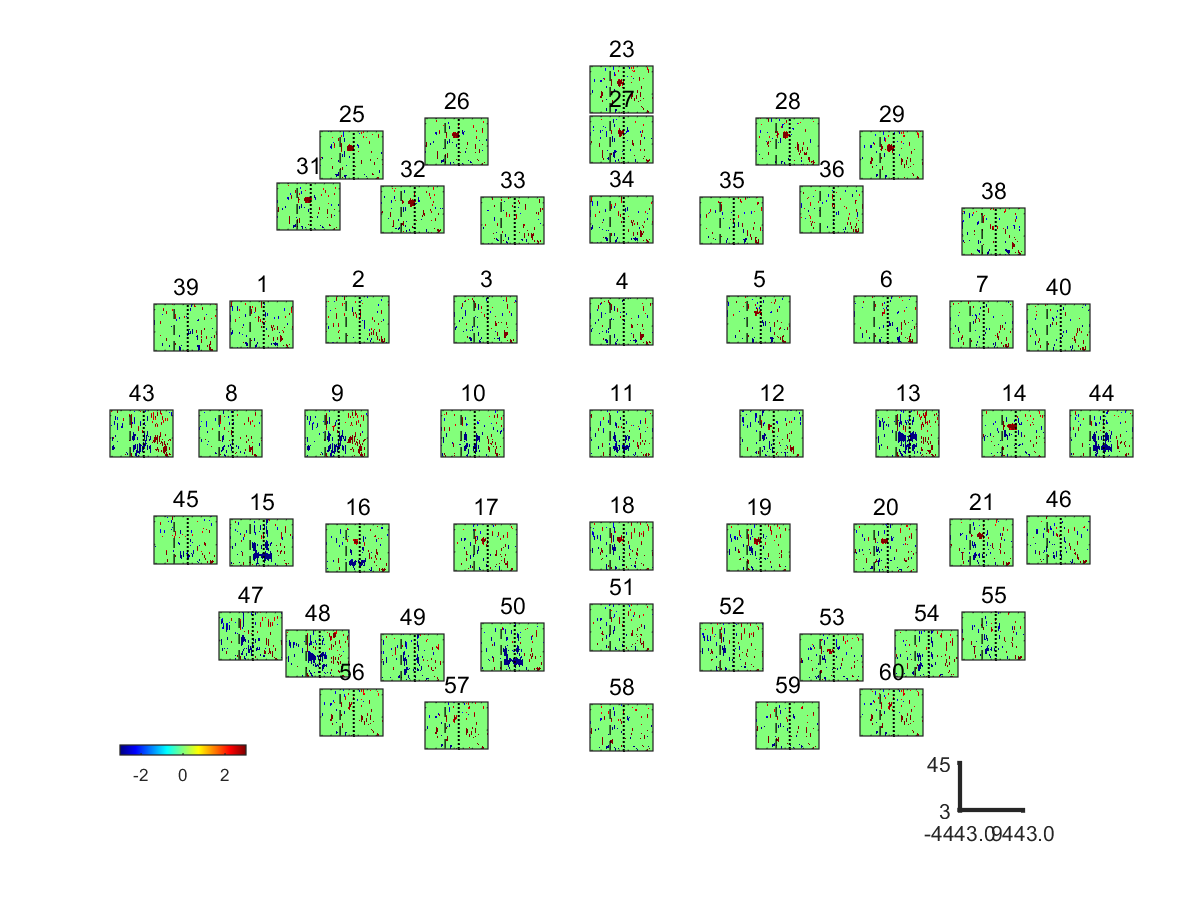


**Supplementary Figure 8.** EEG event related spectral power bootstrap analysis of **S4**: ORTHOSIS vs. REST (Top) and ORTHOFES vs. REST (Bottom)

| Frequency | Conditions | Phase II and III | Phase IV and V |
| --- | --- | --- | --- |
| Alpha  Band | ORTHOSIS  Vs  REST | 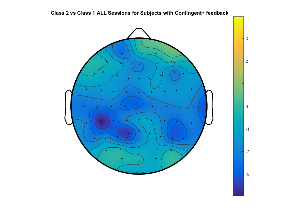 | 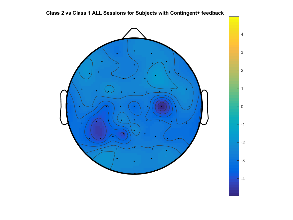 |
|  | ORTHOFES  Vs  REST | 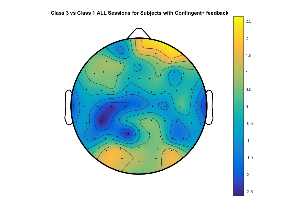 | 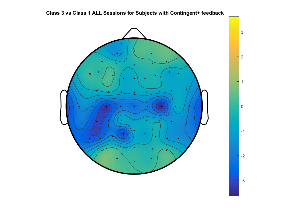 |
| Beta  band | ORTHOSIS  Vs  REST | 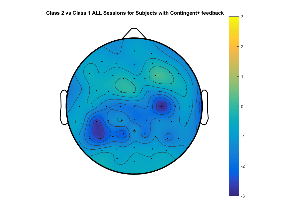 | 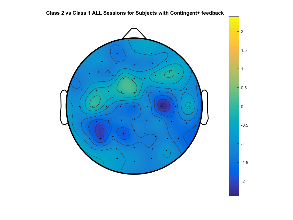 |
|  | ORTHOFES  Vs  REST | 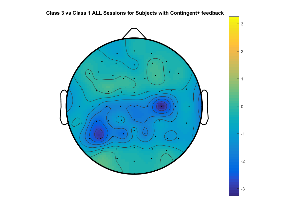 | 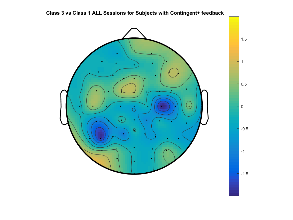 |

**Supplementary Figure 9.**. Averaged differential power values of **S4**. Topography presenting the power changes in ORTHOSIS and ORTHOFES. The 6 ~ 14 Hz and 17 ~ 25 Hz were selected for Alpha and Beta band respectively. The power changes in ORTHOSIS and ORTHOFES were compared to REST. The color bars were autoscaled to detect the meaningful area between conditions and electrodes. Yellow indicates ERS (power increase in dB), and blue shows ERD (power decrease in dB) compared with REST, as expressed in the color bar.


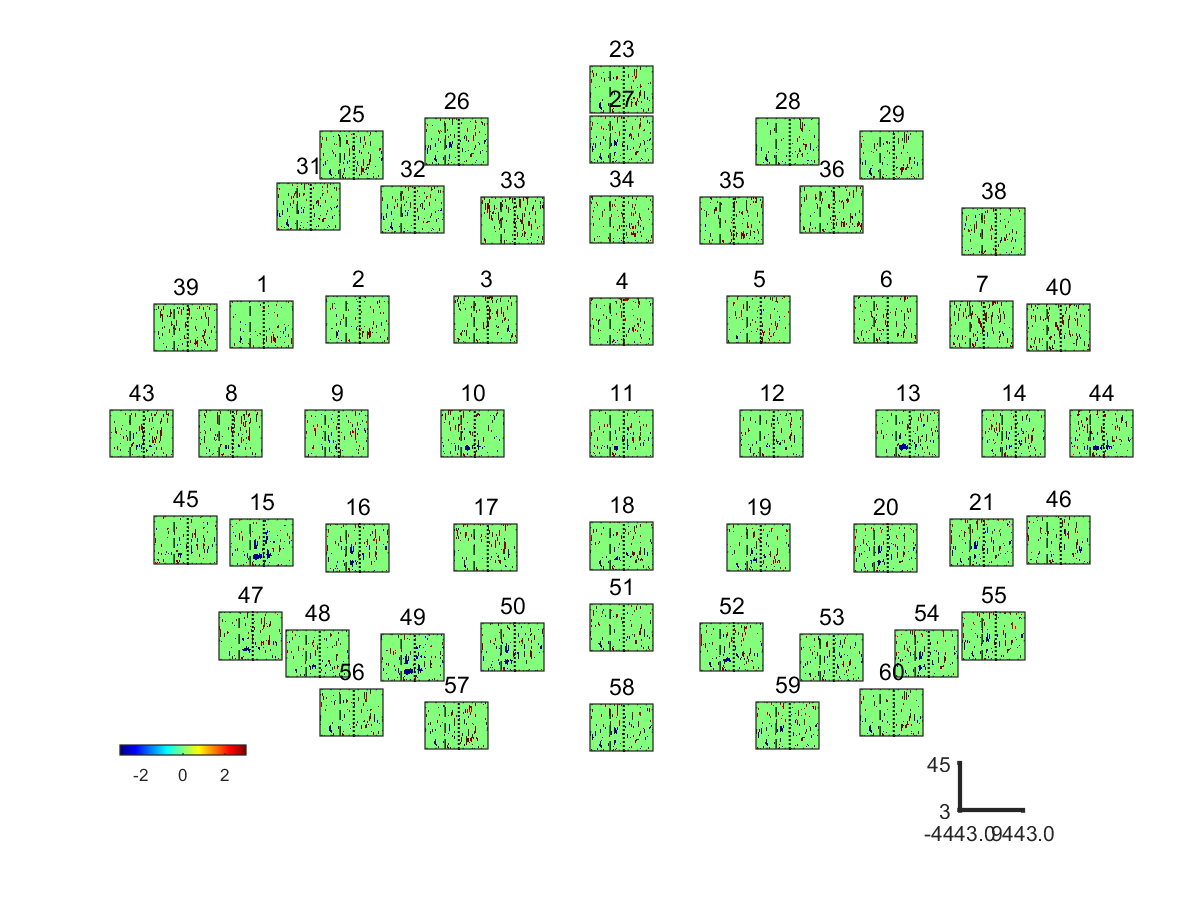

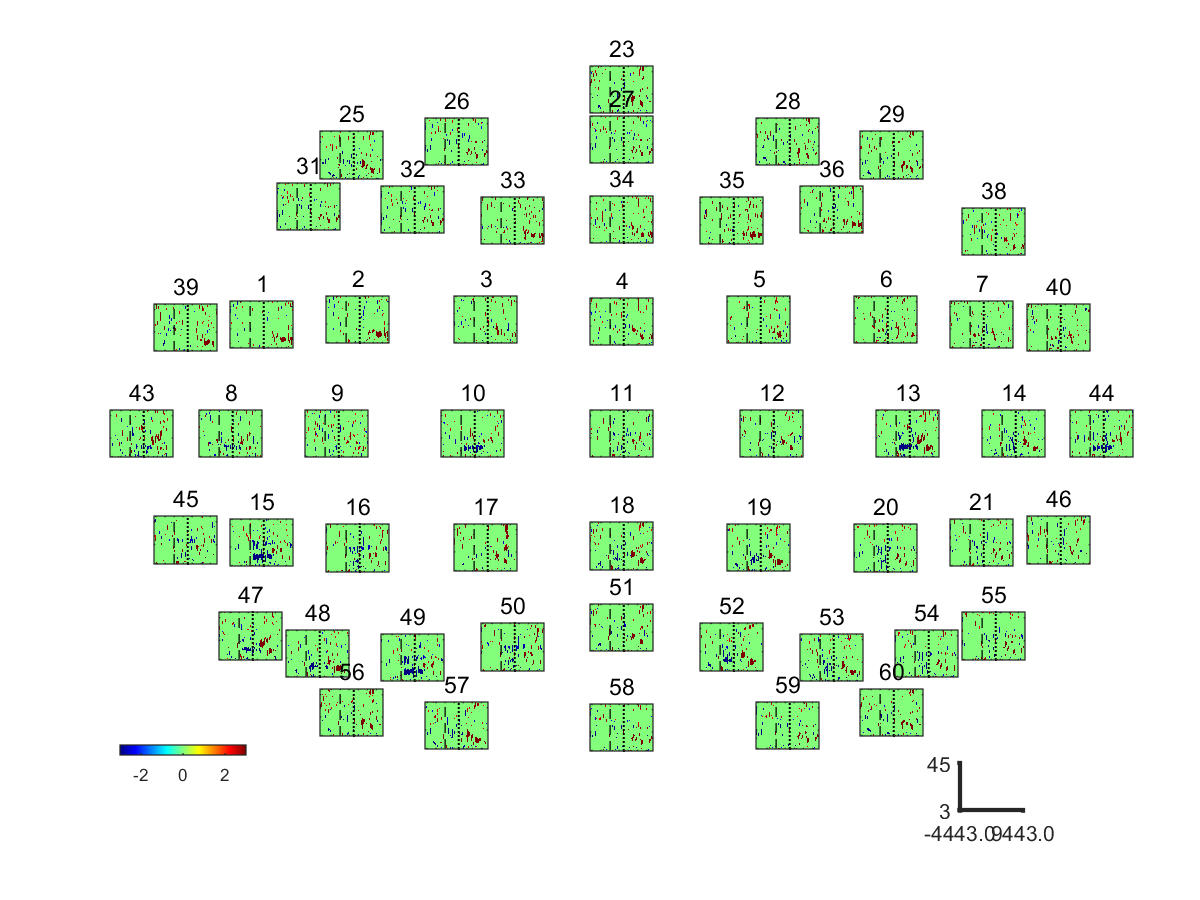


**Supplementary Figure 10.** EEG event related spectral power bootstrap analysis of **S5**: ORTHOSIS vs. REST (Top) and ORTHOFES vs. REST (Bottom)

| Frequency | Conditions | Phase II and III | Phase IV and V |
| --- | --- | --- | --- |
| Alpha  Band | ORTHOSIS  Vs  REST | 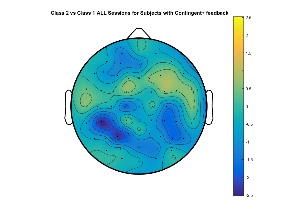 | 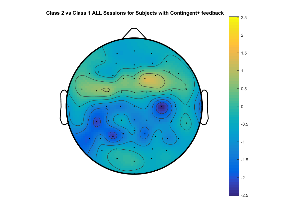 |
|  | ORTHOFES  Vs  REST | 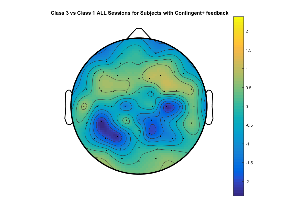 | 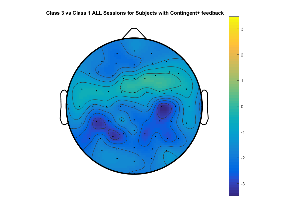 |
| Beta  band | ORTHOSIS  Vs  REST | 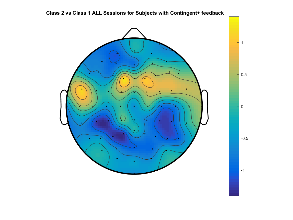 | 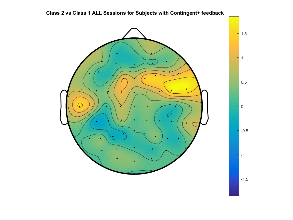 |
|  | ORTHOFES  Vs  REST | 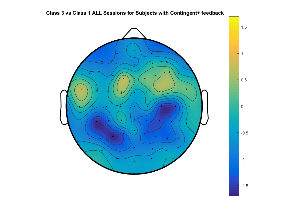 | 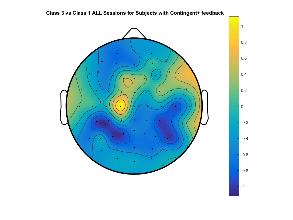 |

**Supplementary Figure 11.**. Averaged differential power values of **S5**. Topography presenting the power changes in ORTHOSIS and ORTHOFES. The 6 ~ 14 Hz and 18 ~ 26 Hz were selected for Alpha and Beta band respectively. The power changes in ORTHOSIS and ORTHOFES were compared to REST. The color bars were autoscaled to detect the meaningful area between conditions and electrodes. Yellow indicates ERS (power increase in dB), and blue shows ERD (power decrease in dB) compared with REST, as expressed in the color bar.


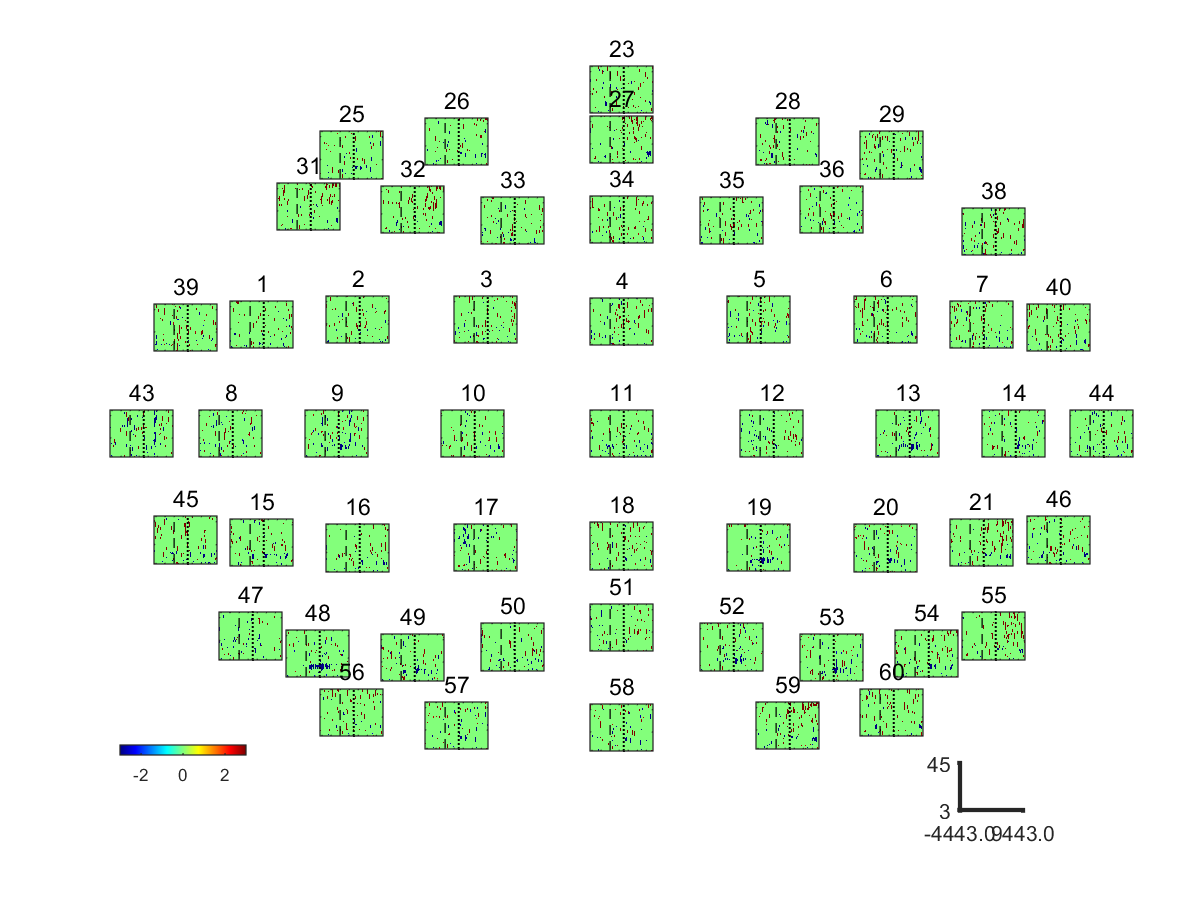

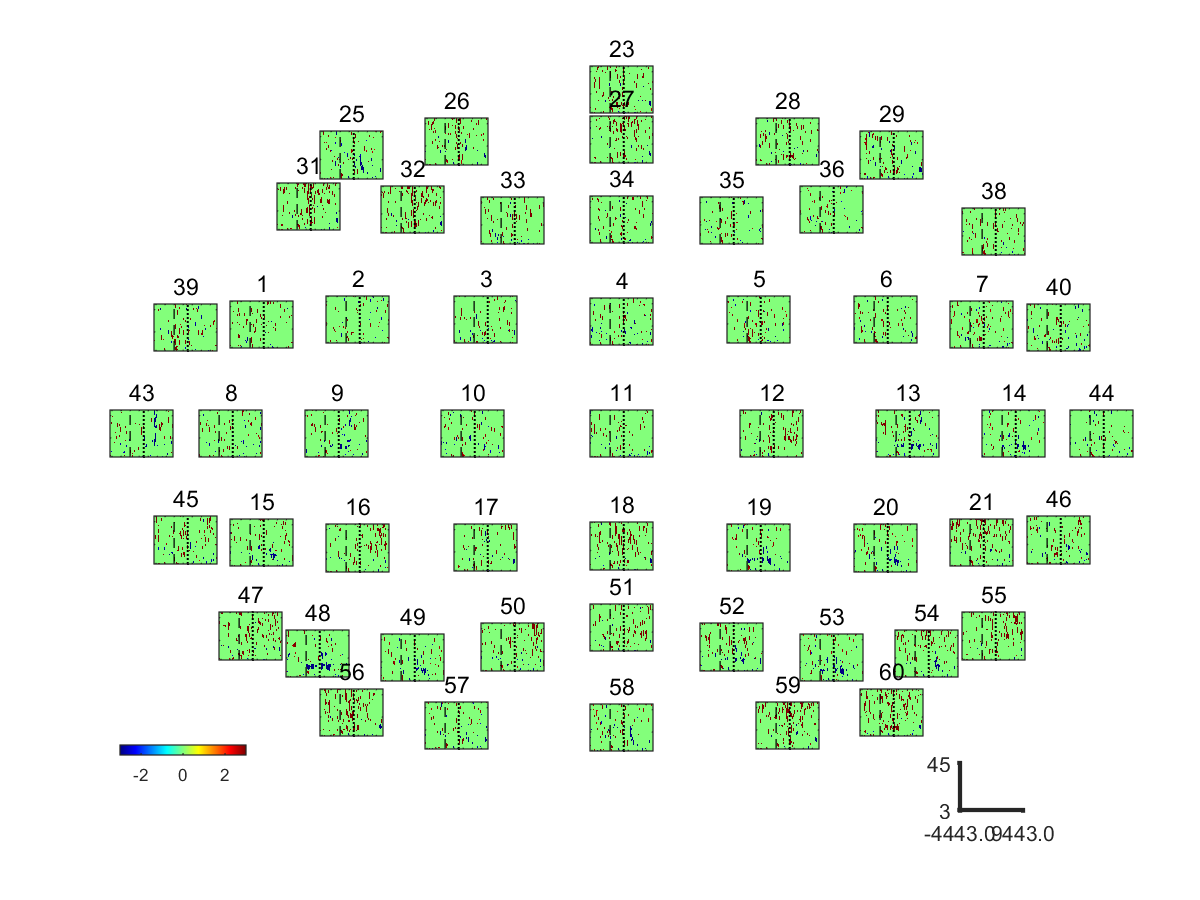


**Supplementary Figure 12.** EEG event related spectral power bootstrap analysis of **S6**: ORTHOSIS vs. REST (Top) and ORTHOFES vs. REST (Bottom)

| Frequency | Conditions | Phase II and III | Phase IV and V |
| --- | --- | --- | --- |
| Alpha  Band | ORTHOSIS  Vs  REST | 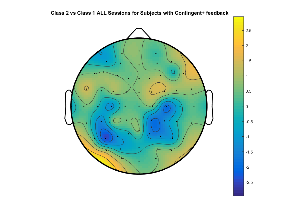 | 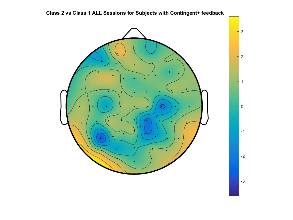 |
|  | ORTHOFES  Vs  REST | 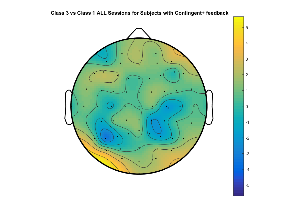 | 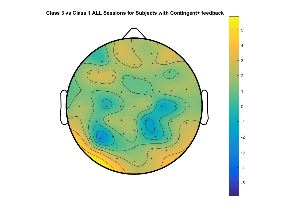 |
| Beta  band | ORTHOSIS  Vs  REST | 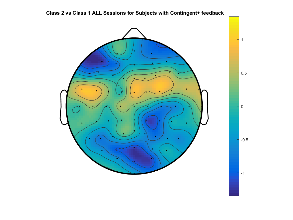 | 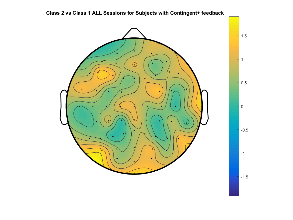 |
|  | ORTHOFES  Vs  REST | 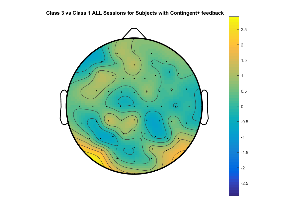 | 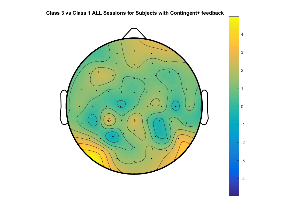 |

**Supplementary Figure 13.**. Averaged differential power values of **S6**. Topography presenting the power changes in ORTHOSIS and ORTHOFES. The 9 ~ 14 Hz and 21 ~ 25 Hz were selected for Alpha and Beta band respectively. The power changes in ORTHOSIS and ORTHOFES were compared to REST. The color bars were autoscaled to detect the meaningful area between conditions and electrodes. Yellow indicates ERS (power increase in dB), and blue shows ERD (power decrease in dB) compared with REST, as expressed in the color bar.


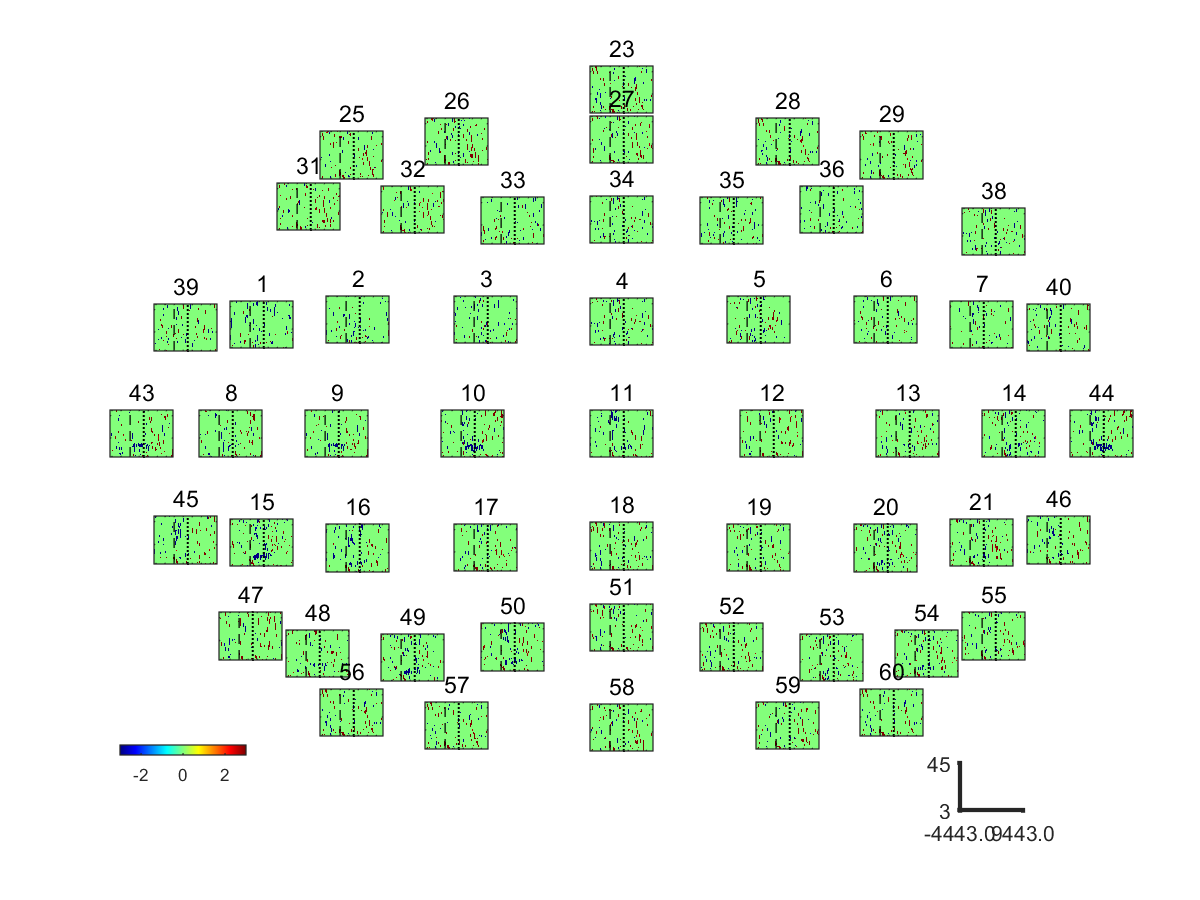


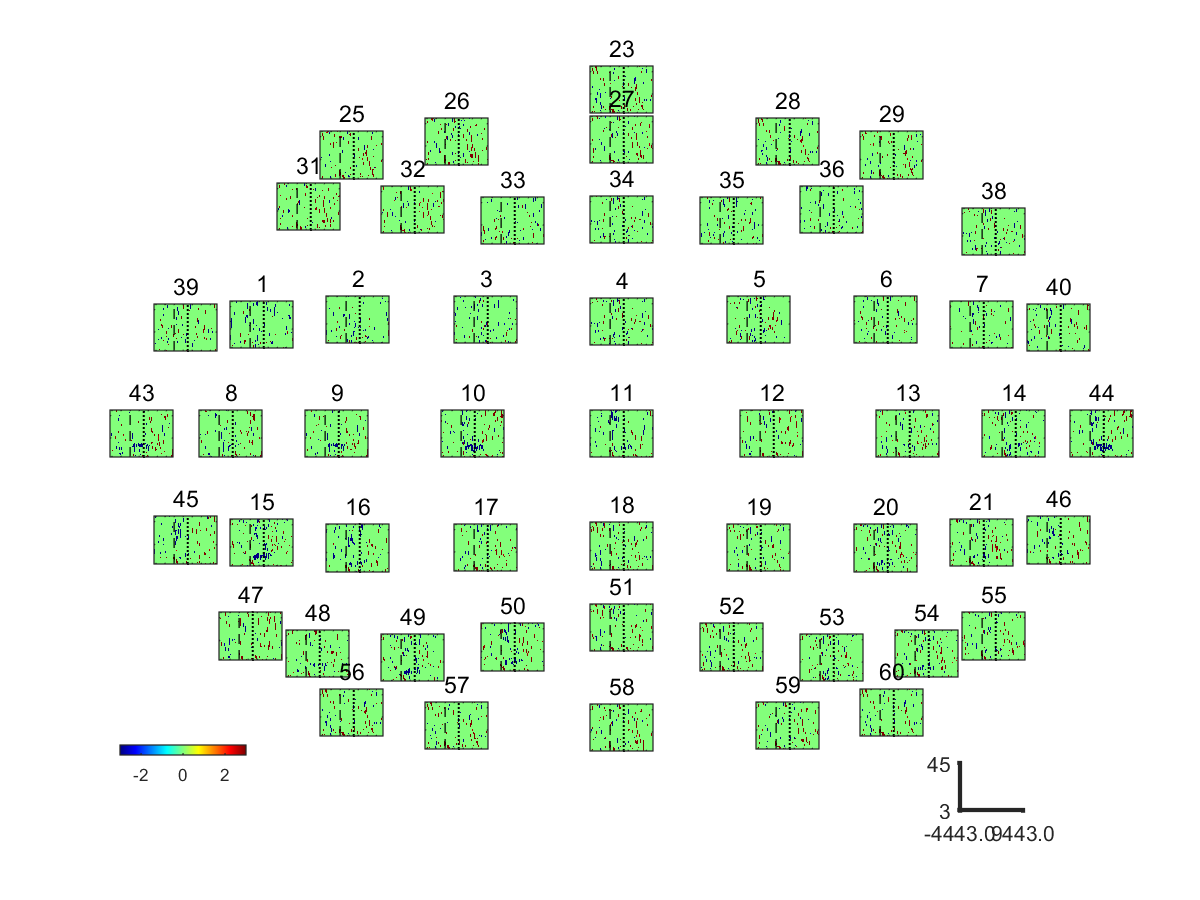


**Supplementary Figure 14.** EEG event related spectral power bootstrap analysis of **S7**: ORTHOSIS vs. REST (Top) and ORTHOFES vs. REST (Bottom)

| Frequency | Conditions | Phase II and III | Phase IV and V |
| --- | --- | --- | --- |
| Alpha  Band | ORTHOSIS  Vs  REST | 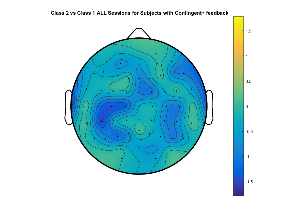 | 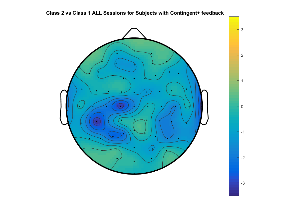 |
|  | ORTHOFES  Vs  REST | 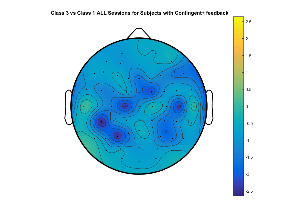 | 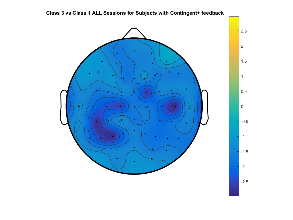 |
| Beta  band | ORTHOSIS  Vs  REST | 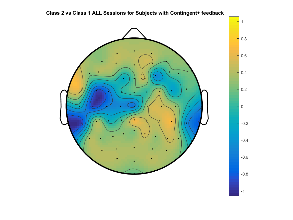 | 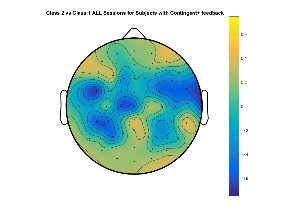 |
|  | ORTHOFES  Vs  REST | 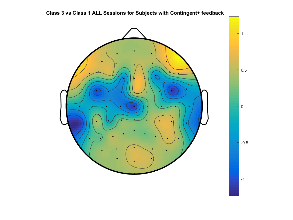 | 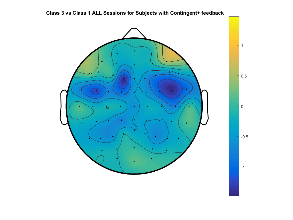 |

**Supplementary Figure 15.**. Averaged differential power values of **S7**. Topography presenting the power changes in ORTHOSIS and ORTHOFES. The 12 ~ 17 Hz and 20 ~ 26 Hz were selected for Alpha and Beta band respectively. The power changes in ORTHOSIS and ORTHOFES were compared to REST. The color bars were autoscaled to detect the meaningful area between conditions and electrodes. Yellow indicates ERS (power increase in dB), and blue shows ERD (power decrease in dB) compared with REST, as expressed in the color bar.


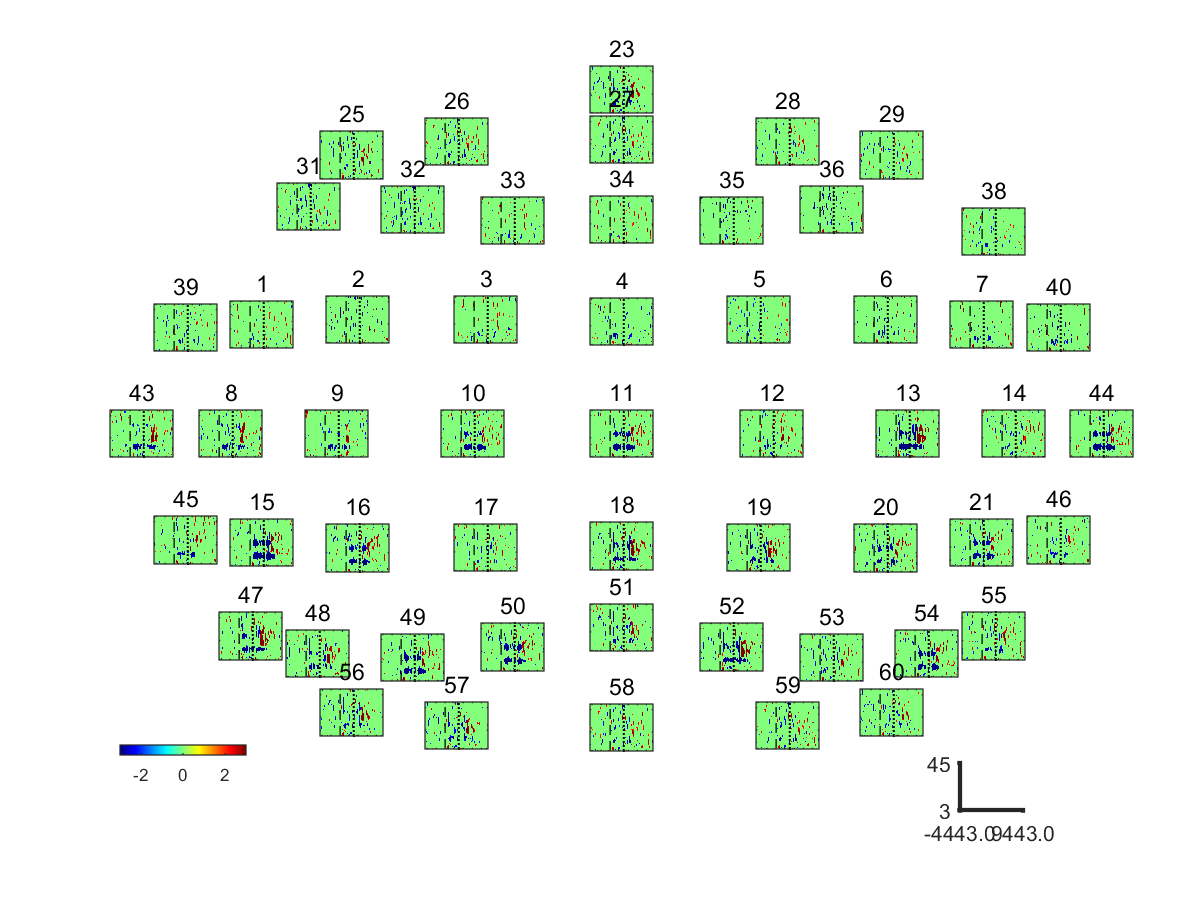

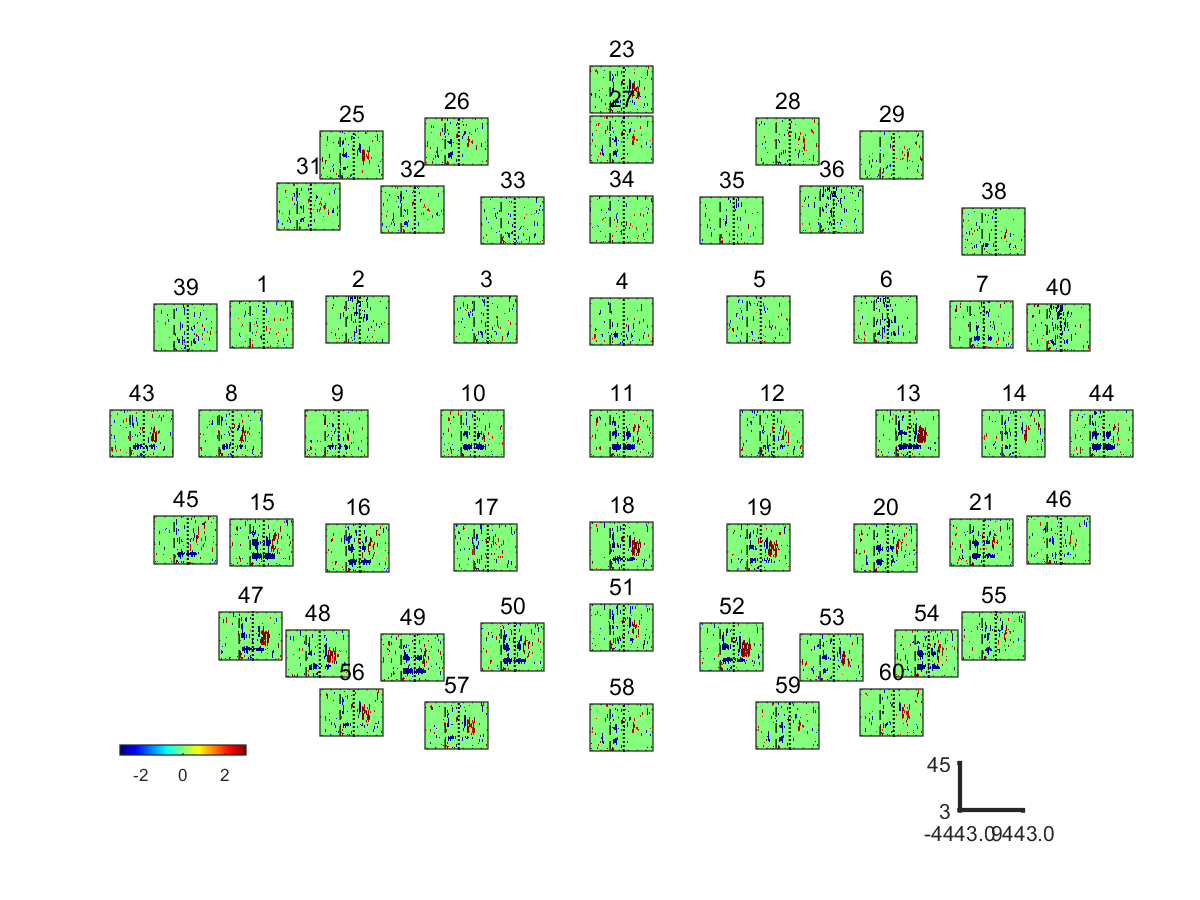


**Supplementary Figure 16.** EEG event related spectral power bootstrap analysis of **S8**: ORTHOSIS vs. REST (Top) and ORTHOFES vs. REST (Bottom)

| Frequency | Conditions | Phase II and III | Phase IV and V |
| --- | --- | --- | --- |
| Alpha  Band | ORTHOSIS  Vs  REST | 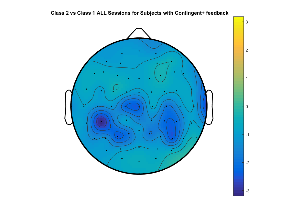 | 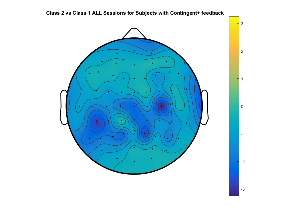 |
|  | ORTHOFES  Vs  REST | 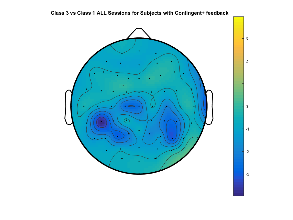 | 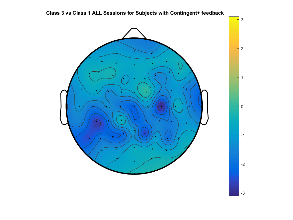 |
| Beta  band | ORTHOSIS  Vs  REST | 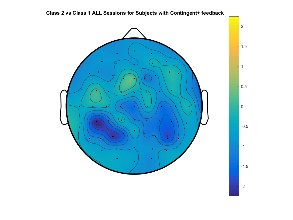 | 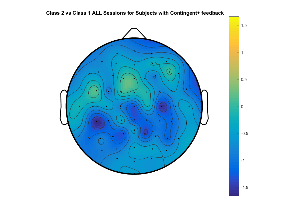 |
|  | ORTHOFES  Vs  REST | 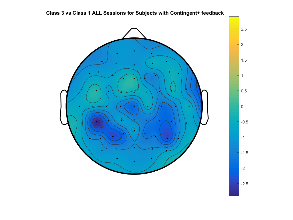 | 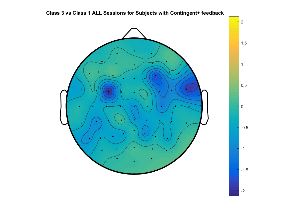 |

**Supplementary Figure 17.**. Averaged differential power values of **S8**. Topography presenting the power changes in ORTHOSIS and ORTHOFES. The 9 ~ 14 Hz and 22 ~ 25 Hz were selected for Alpha and Beta band respectively. The power changes in ORTHOSIS and ORTHOFES were compared to REST. The color bars were autoscaled to detect the meaningful area between conditions and electrodes. Yellow indicates ERS (power increase in dB), and blue shows ERD (power decrease in dB) compared with REST, as expressed in the color bar.


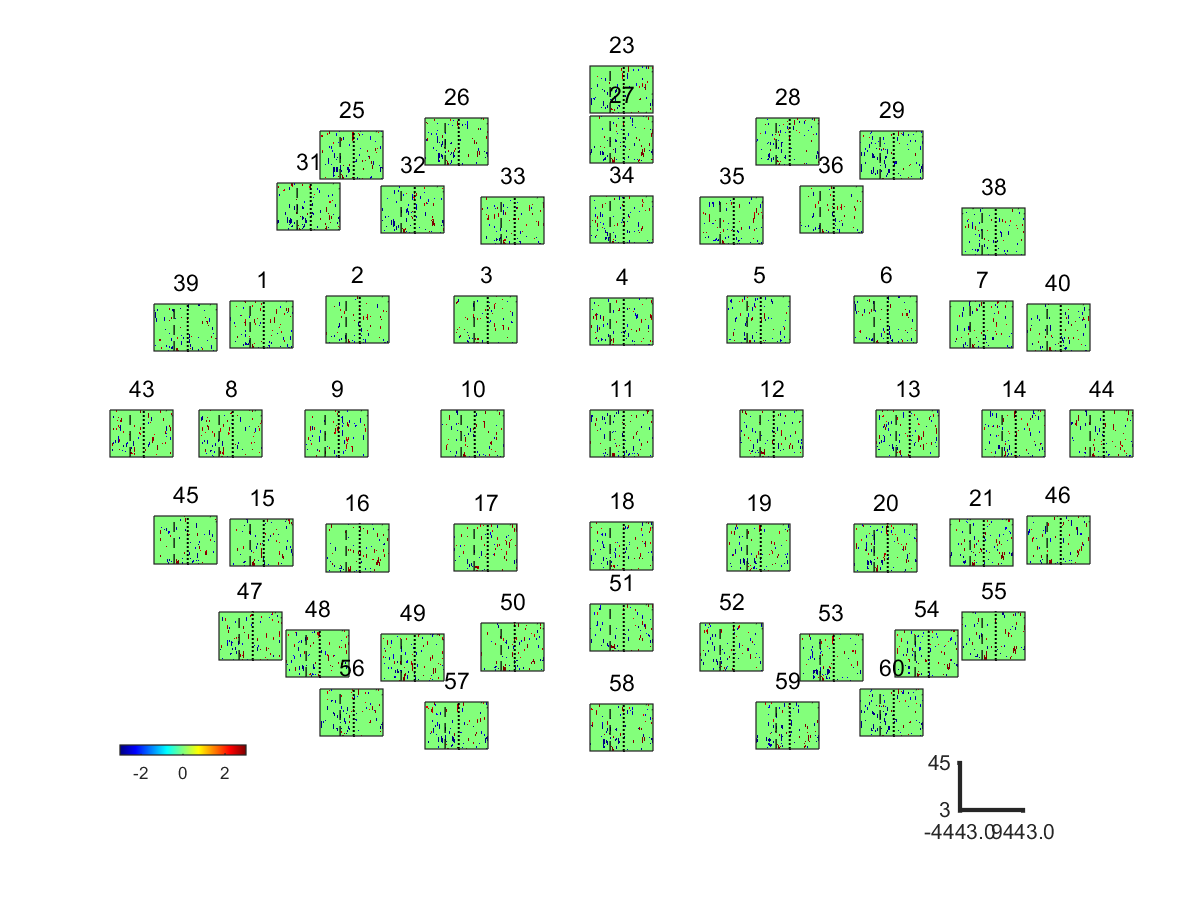


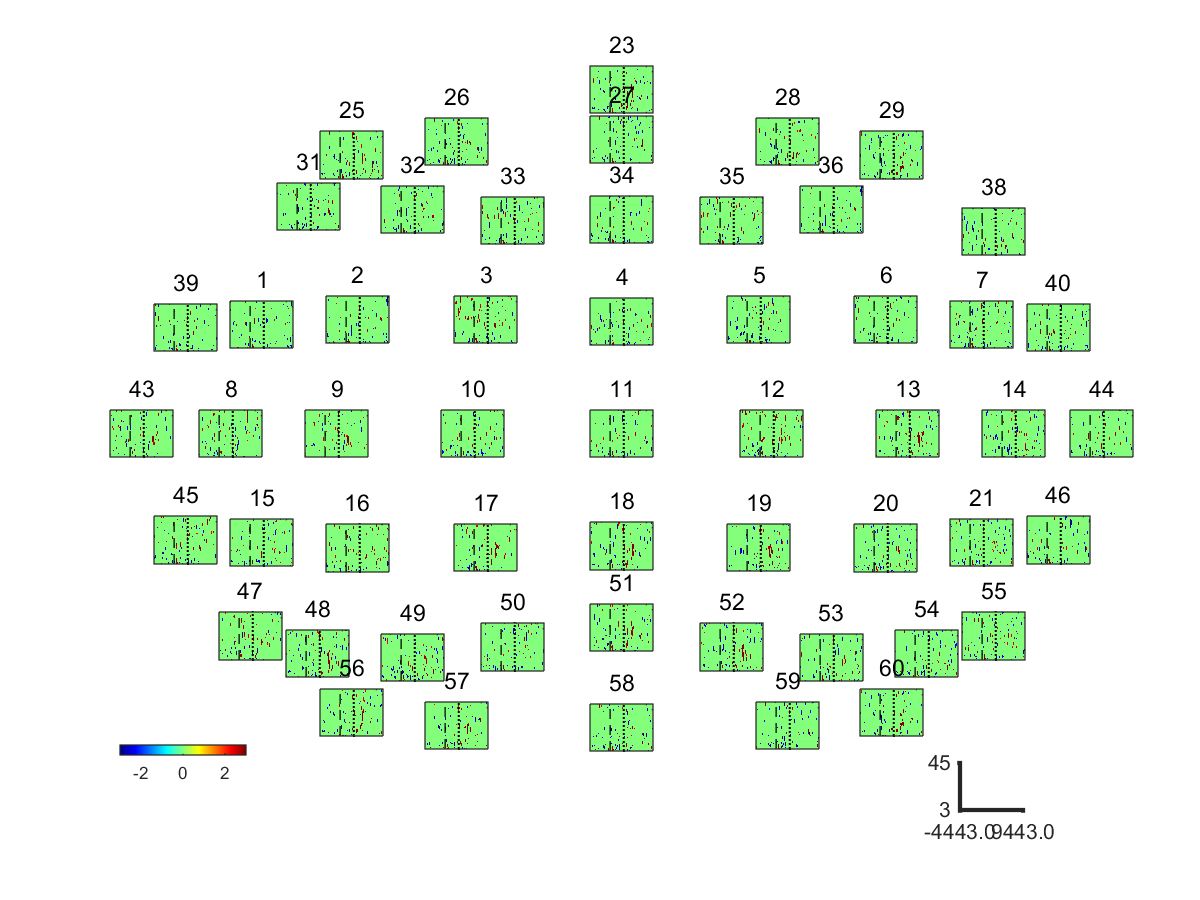


**Supplementary Figure 18.** EEG event related spectral power bootstrap analysis of the **subject excluded (SE1)**: ORTHOSIS vs. REST (Top) and ORTHOFES vs. REST (Bottom)


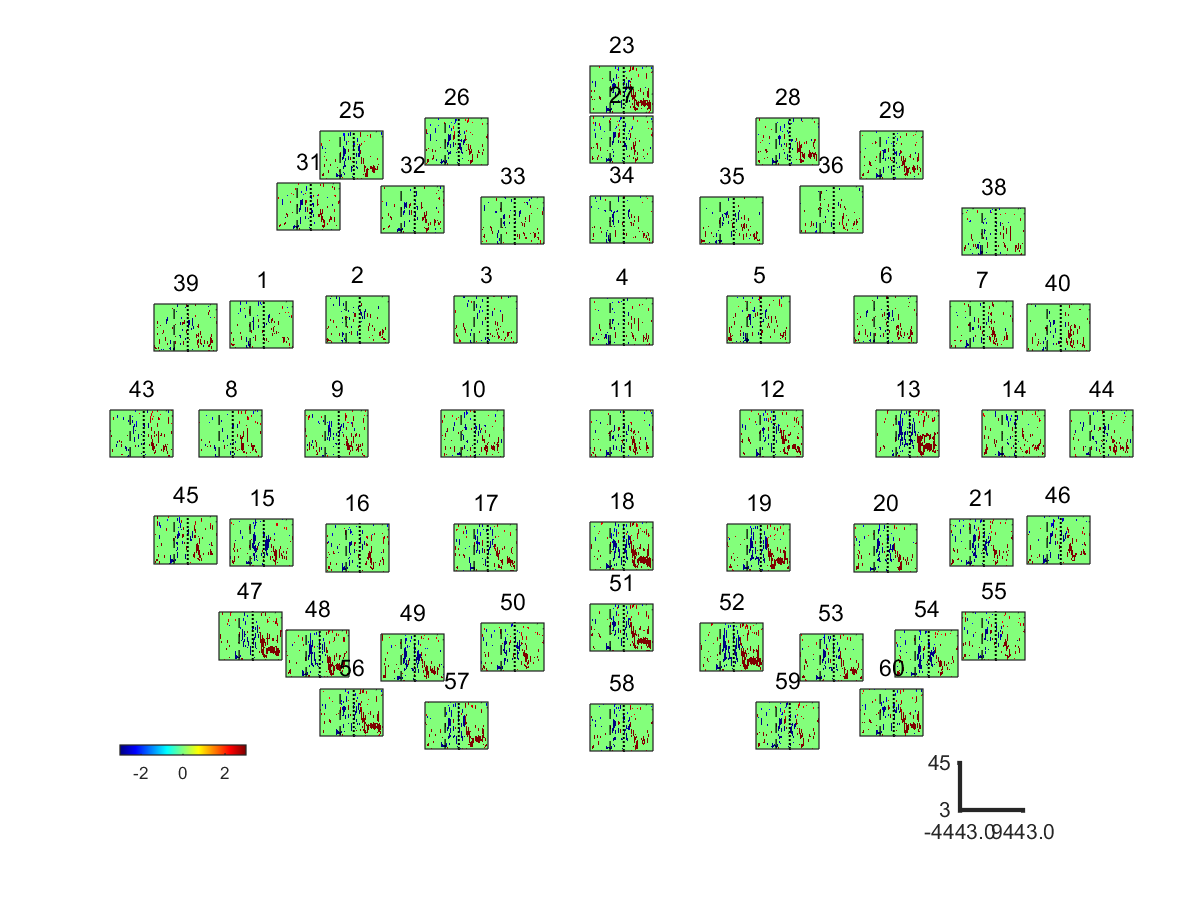


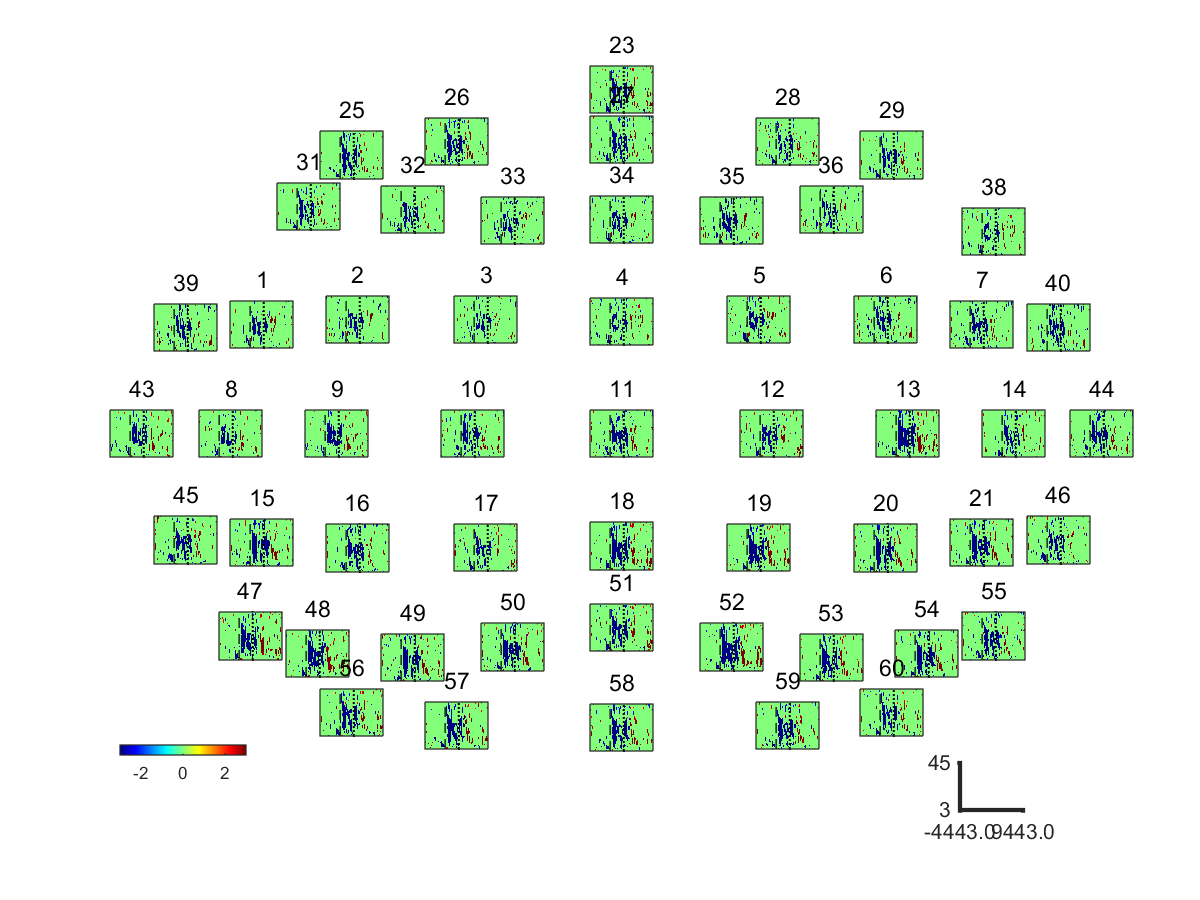


**Supplementary Figure 19.** EEG event related spectral power bootstrap analysis of the **subject excluded (SE2)**: ORTHOSIS vs. REST (Top) and ORTHOFES vs. REST (Bottom)

**
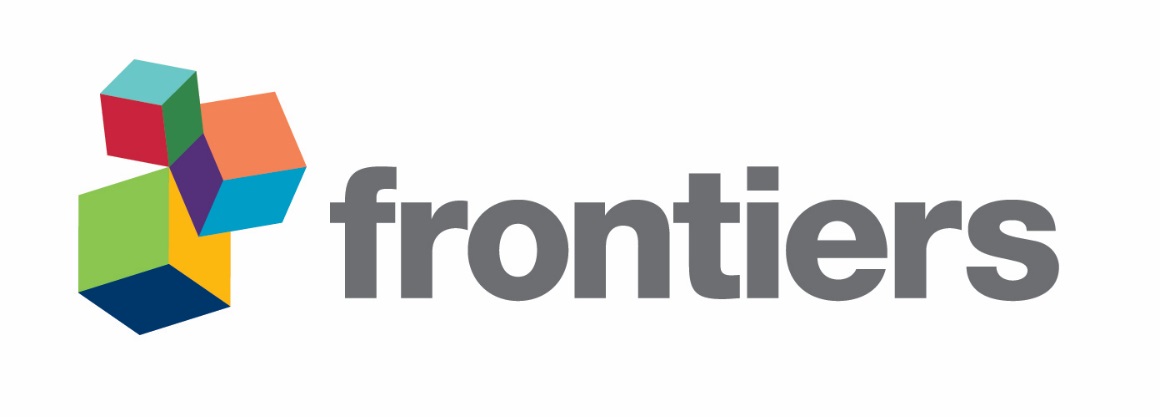
**
